# αv Integrins combine with LC3 and atg5 to regulate Toll-like receptor signalling in B cells

**DOI:** 10.1038/ncomms10917

**Published:** 2016-03-11

**Authors:** Mridu Acharya, Anna Sokolovska, Jenny M. Tam, Kara L. Conway, Caroline Stefani, Fiona Raso, Subhankar Mukhopadhyay, Marianela Feliu, Elahna Paul, John Savill, Richard O. Hynes, Ramnik J. Xavier, Jatin M. Vyas, Lynda M. Stuart, Adam Lacy-Hulbert

**Affiliations:** 1 Immunology Program, Benaroya Research Institute, 1201 Ninth Avenue, Seattle, Washington 98101, USA; 2 Laboratory of Developmental Immunology, Department of Pediatrics, Massachusetts General Hospital/ Harvard Medical School, 55 Fruit Street, Boston, Massachusetts 02114, USA; 3 Division of Infectious Disease, Department of Medicine, , Massachusetts General Hospital/ Harvard Medical School, 55 Fruit Street, Boston, Massachusetts 02114, USA; 4 Gastrointestinal Unit and Center for the Study of Inflammatory Bowel Disease, Massachusetts General Hospital/ Harvard Medical School, 55 Fruit Street, Boston, Massachusetts 02114, USA; 5 Microbial Pathogenesis Group, Wellcome Trust Sanger Institute, Hinxton, Cambridge CB10 1SA, UK; 6 MRC/University of Edinburgh Centre for Inflammation Research, 47 Little France Crescent, Edinburgh EH16 4TJ, UK; 7 Howard Hughes Medical Institute, Koch Institute of Integrated Cancer Biology, Massachusetts Institute for Technology, Cambridge, 500 Main Street, Cambridge, Massachusetts 02139, USA; 8 Bill and Melinda Gates Foundation, 500 Fifth Avenue N, Seattle, Washington 98109, USA

## Abstract

Integrin signalling triggers cytoskeletal rearrangements, including endocytosis and exocytosis of integrins and other membrane proteins. In addition to recycling integrins, this trafficking can also regulate intracellular signalling pathways. Here we describe a role for αv integrins in regulating Toll-like receptor (TLR) signalling by modulating intracellular trafficking. We show that deletion of αv or β3 causes increased B-cell responses to TLR stimulation *in vitro*, and αv-conditional knockout mice have elevated antibody responses to TLR-ligand-associated antigens. αv regulates TLR signalling by promoting recruitment of the autophagy component LC3 (microtubule-associated proteins 1 light chain 3) to TLR-containing endosomes, which is essential for progression from NF-κB to IRF signalling, and ultimately for traffic to lysosomes where signalling is terminated. Disruption of LC3 recruitment leads to prolonged NF-κB signalling and increased B-cell proliferation and antibody production. This work identifies a previously unrecognized role for αv and the autophagy components LC3 and atg5 in regulating TLR signalling and B-cell immunity.

## Abstract (web summary)

Integrins can regulate antigen-specific and innate immune receptor signalling, thereby affecting immune cell function. Here the authors show that avß3 integrin controls Toll-like receptor (TLR) signalling by regulating its trafficking to limit TLR-mediated B-cell proliferation and antibody production.

## Graphical abstract

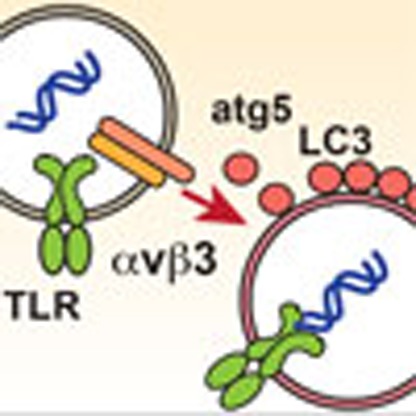

Integrins are heterodimeric membrane proteins that link extracellular signals to the cytoskeleton^1^. αv Integrins comprise the αv subunit paired with one of five different β subunits, and represent a major family of integrins with overlapping specificities and functions^2^. We and others have shown that αv integrins have critical roles in the immune system which include promoting lymphocyte migration^3^,^4^, internalizing and presenting microbial and self-derived ligands to innate immune receptors^5^,^6^,^7^,^8^,^9^ and mediating activation of transforming growth factor (TGF)-β^10^,^11^,^12^,^13^. Many of these functions of αv integrins can be attributed directly to their ability to bind specific ligands and mediate cell–matrix or cell–cell interactions. However αv integrins have also been reported to regulate signalling by other receptor pathways, such as epidermal growth factor and vascular endothelial growth factor receptors, through less well-understood mechanisms. Again, these roles of αv integrins are also implicated in the immune system, where αvβ3 and αvβ5 can promote signalling through antigen-specific and innate immune receptors^8^,^14^, as well as responses to growth factors and chemokines^15^. In B cells, these functions of αv may support differentiation of immature cells and promote lymphoma^15^,^16^.

This property of αv integrins to fine-tune intracellular signalling pathways led us to investigate their role in B-cell responses. Activation of B cells is initiated by antigen binding to diverse, clonally expressed B-cell receptors (BCRs), but full differentiation into antibody producing cells, requires additional accessory signals which determine the type and strength of antibody response. These accessory signals were thought to derive primarily from T-cell co-stimulation, from additional receptors recognizing opsonizing complement and antibody, and from the local cytokine environment. More recently, it has become clear that B-cell activation is also influenced by signals associated with the antigen itself, notably through germline-encoded receptors for microbial antigens, such as, Toll-like receptors (TLRs)^17^. During primary immune responses, these additional signals augment BCR-derived signals to promote proliferation, class switching and entry into germinal centres, ensuring the antibody response is tailored to the source and context of the antigen. In a number of mature B-cell populations, most notably ‘innate B cells' that reside in the marginal zone (MZ) or serosal cavities (B-1 cells), but also in memory and plasma cells, TLR signalling is sufficient to stimulate proliferation and antibody production without strong BCR stimulation^18^,^19^,^20^, ensuring maintenance of a long-lived memory pool in the absence of antigen^21^ and allowing rapid polyclonal responses to neutralize nascent infections^22^. However, TLRs also recognize host-derived components, notably DNA and RNA (ligands for TLR9 and TLR7, respectively), and self ligand activation of TLRs is increasingly implicated in autoreactive cell expansion and autoimmune pathology^23^,^24^,^25^. This is particularly apparent in systemic lupus erythematosus (SLE), where in mouse models, production of characteristic anti-nuclear autoantibodies is directly dependent on B-cell TLR signalling^26^. The potential pathogenic consequences of activation by self-derived ligands therefore requires BCR and TLR signalling to be tightly regulated. Identifying the mechanisms by which this happens is critical for understanding how B cells balance the need for robust defence against pathogens while preventing excessive immune responses to self.

Here we describe a cell-intrinsic role for αv integrins in regulation of B-cell signalling. We report that deletion of αv from B cells leads to expansion of transitional and MZ B cells, and increased antibody responses to self and foreign antigens associated with TLR ligands, and show that in the absence of αv or its partner β3, B cells respond more strongly to TLR ligands. Specifically, we show that αv promotes the recruitment of the autophagy component LC3 (microtubule-associated proteins 1 light chain 3) to TLR-containing endosomes. The effect of this is two-fold: first, LC3 recruitment is required for transition from NF-κB to interferon regulatory factor 7 (IRF7) signalling; and second, TLRs traffic from endosomes to a lysosomal compartment where signalling is terminated. Deletion of αv, LC3b or atg5 lead to prolonged NF-κB and MAPK signalling, and delayed or absent IRF7 activation, causing increased B-cell proliferation and antibody production. Taken together these data identify a new pathway of regulation of B-cell activation that involves αvβ3 and autophagy components, and provide mechanistic insights into the role of αv integrins in regulating immune signalling.

## Results

### αvβ3 is expressed at high levels on activated B cells

**Figure 1:**
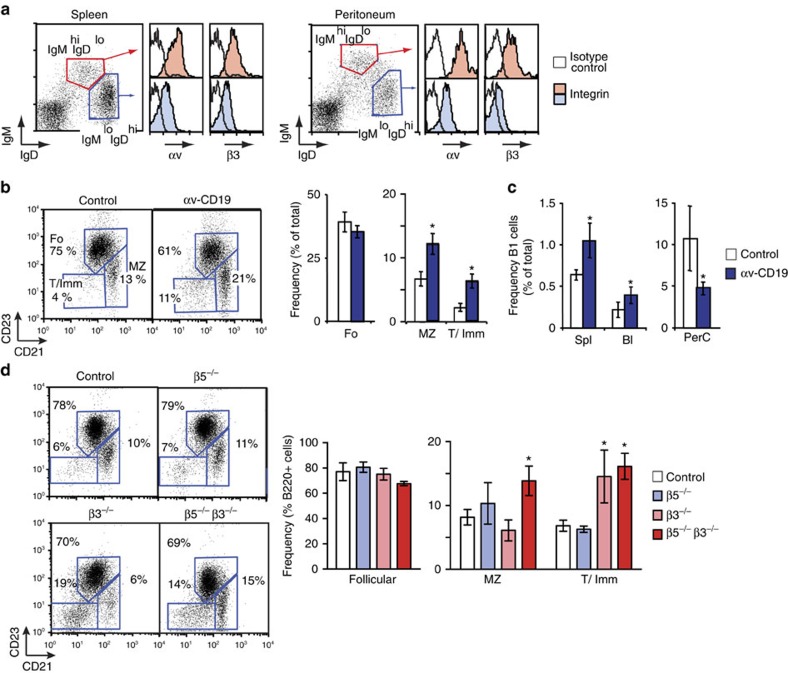
αvβ3 is upregulated in MZ and B-1 cells. (**a**) Surface expression of αv and β3 integrins on spleen and peritoneal B cells. Histograms show staining for αv or β3 (filled histograms) and isotype control antibody (open histograms) on B cells gated based on IgM and IgD expression as indicated. (**b**,**c**) Increased frequency of MZ and B-1 B cells in αv-CD19 mice. (**b**) Representative FACS analysis of splenocytes, gated on B220^+^ cells, and showing regions defining transitional/immature (T/Imm), MZ and follicular (Fo) B cells, and percentages of B220^+^ cells within each region. Mean frequency±s.d. from 4 mice per genotype is shown in histograms. (**c**) Frequency of B-1 cells (B220^+^ CD21^+^ CD5^+^ cells) in spleen, blood and peritoneal cavity of αv-CD19 and control mice. Mean frequency±s.d. from 4 mice per genotype is shown in histograms. (**d**) Representative FACS analysis of splenocytes from β3^−/−^, β5^−/−^, β3β5^−/−^ and control mice, analysed as in (**b**). Mean frequency±s.d. from 3 to 7 mice per genotype is shown in histograms. *P<0.05, student's *t*-test. For all data shown, similar results were seen in three independent experiments.

Developing and mature B cells in bone marrow, lymphoid organs and the peritoneal cavity express surface αv integrin, and one of its partner β subunits, β3, and levels of both integrins are generally higher in IgM^high^ IgD^low^ B-cell populations than in IgD^high^ IgM^low^ cells (Fig. 1; Supplementary Fig. 1). IgM^high^ cells included two related populations of cells, MZ and B-1 B cells, and increased expression of αvβ3 in these cells was confirmed by additional fluorescence-activated cell sorting (FACS) analysis (Supplementary Fig. 1). Lack of suitable antibodies prevented staining for other αv heterodimers by flow cytometry, but RT-PCR (PCR with reverse transcription) demonstrated that B cells also express RNA for β1 and β5 (*Itgb1* and *Itgb5*, respectively) but not β6 and β8 (Supplementary Fig. 1).

B-cell-specific αv-knockout mice were generated (by crossing *Itgav*^flox/flox^ (ref. 10) and *Cd19-Cre* mice^27^). These mice, referred to here as αv-CD19 mice, showed specific and efficient deletion of αv integrins in B cells (described previously in ref. 10 and Supplementary Fig. 1). Early B-cell development appeared largely normal, which is expected as the CD19-Cre transgene does not delete efficiently until late in development, and mature populations of B cells were present in lymph organs and bone marrow of adult mice. However, αv deletion affected transitional, MZ and B-1 cells, and frequencies of all three were increased in spleens of αv-CD19 mice. Similar increases in transitional cells were seen in β3^−/−^ mice, and both transitional and MZ B cells were increased in β3^−/−^ β5^−/−^ double knockout mice. We therefore concluded that αvβ3, and to a lesser extent αvβ5, contribute to MZ and B-1 B-cell numbers in the spleen. αv-CD19 mice also exhibited an increase in B-1 cells in the blood but had decreased numbers in the peritoneal cavity (Fig. 1c). This apparent discrepancy between spleen and peritoneal B-1 cell effects may be explained by increased activation of these cells; in the spleen, this would be expected to cause expansion of cells and re-localization within the spleen, whereas activation of peritoneal B-1 cells causes their exit to the intestine and other sites.

### Increased TLR responses in αv-deficient B cells

**Figure 2:**
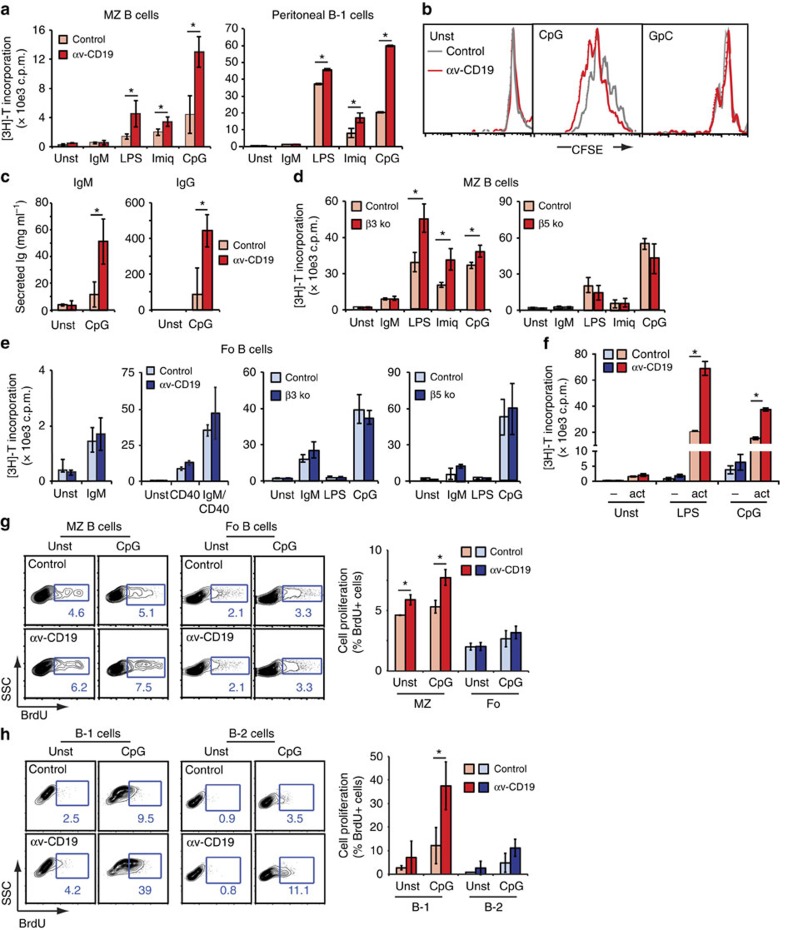
αv regulates TLR response in B cells. (**a**) Proliferation of sorted spleen MZ and peritoneal B-1 B cells populations stimulated in culture with anti-IgM antibody or TLR ligands, measured by [^3^H]-thymidine incorporation. (**b**) FACS histograms of CFSE-labelled spleen cells from indicated mice, gated on MZ B cells, 3 days after stimulation with CpG and control oligonucleotide GpC. (**c**) Immunoglobulin production by peritoneal B cells stimulated with CpG DNA for 3 days, measured by ELISA. (**d**) Proliferation of sorted MZ B cells from β3 and β5 integrin-knockout mice stimulated in culture with anti-IgM antibody or indicated TLR ligands. Proliferation was measured at 72 h of culture by [^3^H]-thymidine incorporation. (**e**) Proliferation of sorted follicular (Fo) B cells from indicated mouse strains stimulated with anti-IgM, anti-CD40 antibodies or TLR ligands, measured by [^3^H]-thymidine incorporation. (**f**) Proliferation of sorted follicular cells left untreated (−) or pre-activated with anti-IgM in culture for 24 h (act), after treatment with TLR ligands. Proliferation was measured by [^3^H]-thymidine incorporation. Data are mean±s.d. of cultures from 3 individual mice per experiment. (**g**,**h**) FACs analysis of spleen MZ/ follicular B cells (**g**), or peritoneal B-1/B-2 cells (**h**) from mice unstimulated or treated with CpG DNA. Representative FACS plots with BrdU^+^ gates and percentage of positive cells are shown. In all cases, histograms show combined data as mean±s.d. from cultures or primary cells from 3 individual mice per group. * represents samples that are significantly different, *P*<0.05, Student's *t*-test and similar results were seen in at least two independent experiments. Unst, unstimulated.

To investigate the role of αv further, subpopulations of primary B cells were sorted from αv-CD19 and control mice, and stimulated in culture. MZ and B-1 cells showed little response to BCR crosslinking, with no difference between control and αv-deficient cells (Fig. 2a). However, when stimulated with TLR ligands, MZ and B-1 cells proliferated robustly, and this was significantly increased in αv-deficient cells compared with controls, regardless of the TLR ligand used (Fig. 2a). This was particularly pronounced for responses to TLR9-stimulating CpG oligonucleotides (CpG), to which all cells in the culture proliferated and was due to TLR signalling as no proliferation was seen in response to the non-TLR ligand control oligonucleotide GpC (Fig. 2b). αv-deficient cells also produced significantly more IgM and IgG after stimulation through TLRs (Fig. 2c). Similar increases in proliferation were seen in β3^−/−^ MZ B cells stimulated through TLRs, whereas β5^−/−^ B cells proliferated normally (Fig. 2d).

Deletion of αv, β3 or β5 had no effect on proliferation of follicular B cells stimulated through crosslinking of the BCR or through the co-stimulatory molecule CD40 (Fig. 2e), suggesting that these integrins were not promoting general survival of proliferating cells, but were specifically affecting response to TLR stimulation. Naive follicular B cells do not respond strongly to TLR stimulation, and the low levels of proliferation seen in response to CpG were unaffected by αv (Fig. 2e). To assess the role of αv in TLR response in follicular cells, we first activated them by BCR crosslinking. This allows these cells to respond strongly to TLR stimulation^19^ (Fig. 2f) but does not affect their expression of surface αv (Supplementary Fig. 2). In this activated state, αv-deficient cells proliferated significantly more than control cells in response to all TLR ligands, as we saw for MZ B cells (Fig. 2f).

αv Deletion similarly promoted B-cell proliferation *in vivo*. Basal proliferation of both MZ and B-1 cells was increased in αv-CD19 mice compared with littermates (as measured by 5-bromo-2-deoxyuridine (BrdU) incorporation; Fig. 2g,h), and in direct agreement with our *in vitro* observations, higher numbers of proliferating MZ and B-1 cells were detected after injection of mice with CpG (Fig. 2g,h). Proliferation of follicular B cells, which are not stimulated robustly by TLR ligands, was unaffected by αv deletion. We therefore concluded that αv regulates B-cell responses to stimulation through TLRs, and that this is mediated by αvβ3.

### Increased antibody responses in αv-CD19 mice

**Figure 3:**
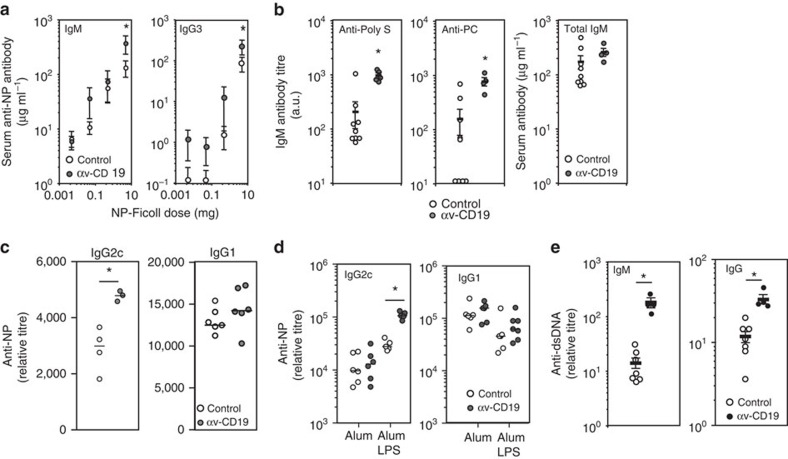
Antibody responses in αv-CD19 mice. (**a**) Serum titres of anti-NP antibody titres in αv-CD19 mice and control mice 5 days following immunization with indicated dose of NP-Ficoll. (**b**) Natural antibody to *S. pneumoniae* polysaccharide (Poly S) and phosphorylcholine (PC) in non-immunized αv-CD19 mice and control mice. Corresponding total IgM amounts for the same mice are also shown. (**c**,**d**) Serum anti-NP IgG2c and IgG1 from αv-CD19 mice and littermate controls 14 days after immunization with NP-CG in combination with either LPS (**c**), alum (**d**) or combined LPS/ alum (**d**). (**e**) Serum anti-dsDNA IgM and IgG antibodies from αv-CD19 mice and littermate controls at 40 weeks of age. Data are shown as either data points from individual mice with mean, or as mean±s.e.m. for at least 4 mice per condition. *Significantly different from control, *P*<0.05, Mann–Whitney–Wilcoxon test. All experiments were repeated at least two times with similar results.

We next analysed antibody responses in αv-CD19 mice. Consistent with the lack of change in total B-cell number in αv-CD19 mice, total serum immunoglobulin levels were similar to those in control mice (Supplementary Fig. 3). However, αv-CD19 mice had 5–10 times higher titres of natural antibody than controls, and produced more antigen-specific IgM and IgG3 following immunization with the T-independent antigen, NP (4-hydroxy-3-nitrophenyl)-Ficoll, (Fig. 3a,b). Natural and T-independent responses are mediated by B-1 and MZ B cells, and these data are therefore consistent with the increased MZ and B-1 cell proliferation in αv-CD19 mice.

As our *in vitro* studies indicated that αv specifically regulated TLR signalling in B cells, we immunized mice with a T-cell-dependent antigen (NP-Chicken gamma globulin (NP-CG)) using the TLR ligand LPS as an adjuvant, αv-CD19 mice produced similar titres of anti-NP IgM and IgG to control mice (Supplementary Fig. 3), but made significantly higher levels of anti-NP IgG2c isotype antibody (Fig. 3c). Previous studies of the role of TLR ligands as adjuvants^28^ have shown that class switching to IgG2 isotypes requires B-cell-intrinsic TLR signalling^29^,^30^. Our data were therefore consistent with effects on LPS signalling to B cells. Supporting this, we found that αv-CD19 mice mounted normal antibody responses when immunized using an alternative adjuvant, alum, which does not act through TLR signalling, whereas combined alum and LPS again led to higher levels of IgG2c (Fig. 3d). B-cell-intrinsic TLR signalling has also been implicated in the production of autoantibodies to nucleic acids in mouse models of SLE^31^,^32^, and anti-dsDNA (double-stranded DNA) autoantibody titres were significantly higher in αv-CD19 mice than littermate controls (Fig. 3e). Together, therefore, these functional studies demonstrate that αv integrins regulate B-cell proliferation and antibody production in response to self and foreign antigens associated with TLR ligands.

### αv Promotes intracellular TLR9 trafficking

The ability of TLRs to signal is regulated by their subcellular localization, which controls both ligand accessibility and the availability of specific signalling adaptors^33^. Signalling through the intracellular TLRs, such as TLR7 and TLR9, first require the TLR and ligand to traffic to the same endosomal compartment and subsequent signalling requires additional trafficking events, with NF-κB and IRF7 activation occurring from distinct endosomal compartments^34^,^35^,^36^. Likewise, although cell surface TLRs such as TLR4 bind ligands and signal through NF-κB at the plasma membrane, they must relocate to endosomal compartments to engage TRIF and promote IRF activation^37^,^38^. Integrins, particularly αvβ3 and α5β1, affect signalling through growth factor receptors by directing their trafficking^39^,^40^ and we therefore decided to follow TLR localization by confocal microscopy to test the hypothesis that αv regulated TLRs through effects on intracellular trafficking. MZ B cells express a number of TLRs (which was unaffected by αv deletion; Supplementary Fig. 4), but we elected to focus on TLR9 for which we had a suitable antibody that would specifically recognize the endogenous protein (as previously reported in ref. 41 and confirmed by us in Supplementary Fig. 5).

**Figure 4:**
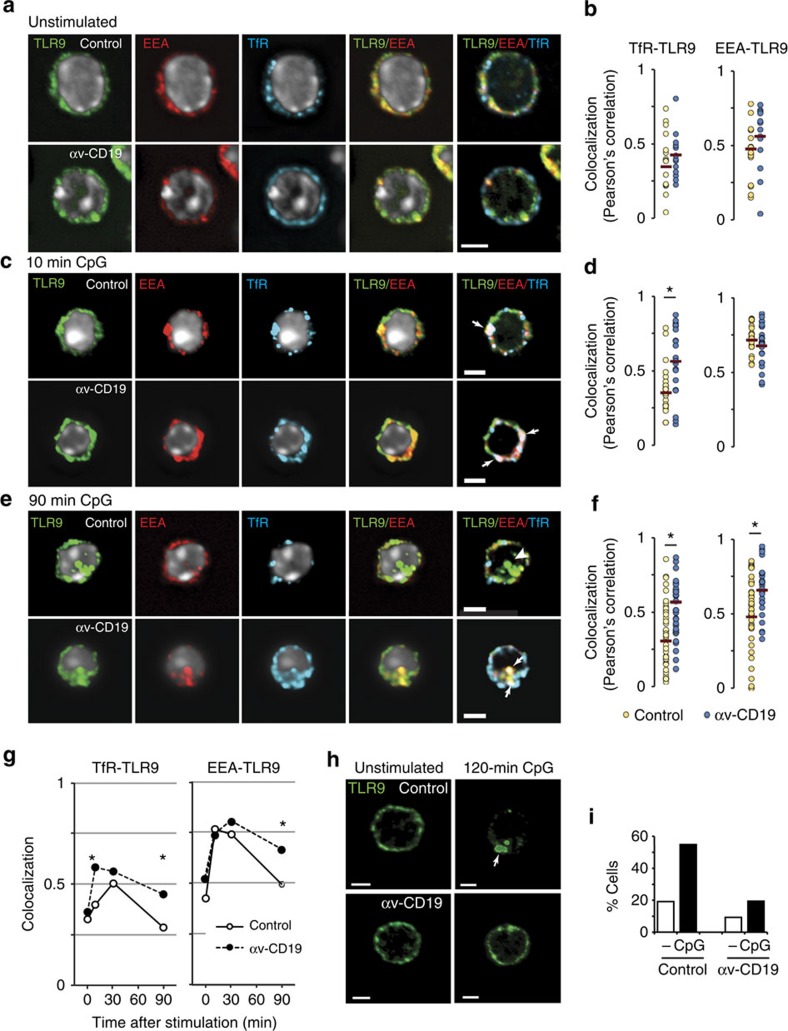
αv regulates intracellular localization of TLR9. (**a**–**f**) Sorted MZ B cells from control or αv-CD19 mice stained with antibodies to TLR9, EEA and Transferrin receptor (TfR). (**a**,**c**,**e**) Representative images from single confocal sections, with Hoechst (white) indicating the cell nucleus. Arrows show regions of TLR9 co-localization with EEA and/or TfR (Hoechst omitted for clarity in these images), and arrowhead in **e** marks area of TLR9 accumulation in control cells that is not associated with TfR or EEA. (**b**,**d**,**f**,**g**) Quantification of co-localization between TLR9 and EEA or TfR. Plots show co-localization values for individual cells and median (**b**,**d**,**f**) or median alone (**g**), for >20 cells per condition. *Significantly different from control, *P*<0.005, Mann–Whitney–Wilcoxon test. (**h**) Sorted MZ B cells from control or αv-CD19 mice stained with antibody against TLR9, with or without stimulation with CpG DNA for 120 min. Representative images are from a single confocal section. Arrow indicates re-localization of TLR9 seen in control cells. (**i**) Quantification of TLR9 re-localization in sorted spleen MZ or peritoneal B-1 B cells. Each data point is based on analysis of at least 30 cells by confocal microscopy. Graph represents percentage of cells with reorganized TLR9 as seen in 120 min control cells in figure (**h**). In all cases, similar results were seen in three independent experiments. Scale bars, 2.50 μm.

In both αv knockout and control unstimulated cells TLR9 showed some co-localization with markers for recycling and early endosomes (transferrin receptor (TfR) and early endosome antigen 1 (EEA1), respectively; Fig. 4a,b) indicating that a small proportion of TLR9 was present in endosomes with the remainder likely to be in endoplasmic reticulum and Golgi^41^,^42^,^43^ or in endo-lysosomes^44^. Following stimulation with CpG DNA, TLR9 co-localization with EEA1 and TfR increased in both control and αv-deficient MZ B cells within 10 min (Fig. 4c,d). By 90 min, TLR9 co-localization with EEA1 and TfR reduced in control cells to levels similar to that in unstimulated cells (Fig. 4e,f). In contrast, in αv-deficient cells, TLR9 co-localization with EEA1 and TfR remained elevated (Fig. 4e–g). Furthermore, by 2 h TLR9 in control cells had accumulated into large intracellular compartments (Fig. 4h), whereas in αv-deficient cells, this did not occur (Fig. 4h,i). We therefore concluded that αv regulates re-localization of TLR9 from early endosomes at later signalling time points (45–120 min).

### αv Promotes co-localization of TLR9 with LC3

**Figure 5:**
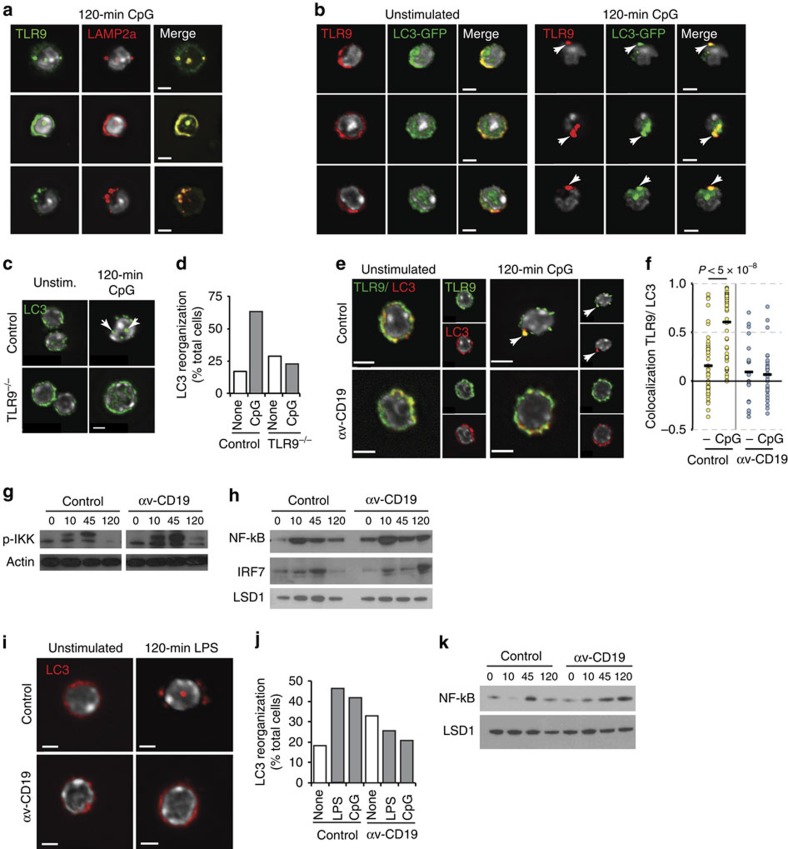
αv regulates TLR signalling by engagement of autophagy machinery. (**a**) Confocal microscopy of TLR9 and Lamp2a staining in sorted MZ B cells from wild-type mice. Hoescht staining shows location of the nucleus (omitted from overlay images for clarity). (**b**) TLR9 staining in GFP-LC3 transgenic mice. Arrows indicate TLR9-LC3 co-localization 120 min after CpG DNA treatment. Representative images are from a single confocal section, and Hoechst (white) indicates the cell nucleus. (**c**) LC3 re-organization measured by antibody staining in sorted MZ B cells from TLR9^−/−^ and wild-type mice before and after incubation with CpG DNA for 120 min. Arrows indicate large aggregations of LC3 in control cells (**d**) Quantification of cells with LC3 aggregation as seen in **c** (from analysis of at least 60 cells/ condition). (**e**) TLR9 and LC3 co-localization measured by antibody staining in sorted MZ B cells from control and αv-CD19 mice. (**f**) Quantification of TLR9 and LC3 co-localization. Each data point represents Pearson's correlation value for a single cell, bars show mean co-localization. *P* value determined by Mann–Whitney–Wilcoxon test. (**g**,**h**) Western blot analysis of phosphorylated IKKα in cytoplasmic fractions (**g**), and NF-κB and IRF7 in nuclear fractions (**h**) from sorted MZ B cells isolated from αv-CD19 and control mice, stimulated with CpG DNA for the indicated time (mins). Also shown are staining of actin (**g**) or LSD1 (**h**) to confirm equivalent protein loading. In both cases, representative blots from at least three independent experiments are shown. (**i**) LC3 staining in peritoneal B cells from control and αv-CD19 after LPS stimulation. (**j**) Quantification of LC3 aggregation after LPS or CPG stimulation in peritoneal B cells based on counting of at least 100 cells per condition. (**k**) Western blot analysis of nuclear NF-κB in sorted MZ cells after LPS stimulation with levels of LSD1 as loading control. All microscopy data are representative of 2–4 independent experiments. Scale bars, 2.50 μm, except **e**, where they show 2.90 μm. For quantification of western analysis and full blots see Supplementary Figs 6 and 7.

At 2 h after stimulation, TLR9 co-localized with lysosome associated membrane protein (LAMP2a) (Fig. 5a), consistent with trafficking to lysosomes. This trafficking of TLR9 was reminiscent of the accumulation of TLR9 in ‘auto-phagosome-like' structures in B cells previously reported by Chaturvedi^41^ and we therefore tested whether TLR9 co-localized with a component of the autophagy machinery and marker of auto-phagosomes, LC3. We observed that TLR9 co-localized with LC3 in primary MZ and B-1 cells, initially in small endosome-like structures ∼45 min after stimulation, and this increased significantly as TLR9 became organized into the larger structures seen 2 h after TLR stimulation (Fig. 5b). Notably, re-localization of LC3 did not occur in TLR9^−/−^ cells, indicating that TLR signalling initiated this process (Fig. 5c,d). αv was critical for this process, as both co-localization of LC3 and TLR9, and the formation of large LC3-positive complexes, were reduced in αv-deficient B cells (Fig. 5e,f).

### Increased and prolonged TLR signalling in αv-deficient cells

Given the critical role of subcellular localization for TLR9 signalling, we predicted that the alterations in trafficking would be associated with changes in activation of NF-κB and IRF pathways. In MZ B cells from control mice, phosphorylation of Iκ-B-kinase (IKK) occurred rapidly (within 10 min) following TLR stimulation and returned to near basal levels by 2 h (Fig. 5g). Likewise, nuclear localization of the p65 component of NF-κB peaked at 10 min and largely subsided by 2 h (Fig. 5h). In contrast, in αv-deficient B cells, the peak levels of both phosphorylation of IKK and nuclear localization of NF-κB were increased following TLR stimulation compared with control cells, and in the case of NF-κB, activation was prolonged and remained elevated at later time points (Fig. 5g,h; Supplementary Fig. 6). We also noted that nuclear localization of NF-κB was higher in αv-deficient B cells than in controls even before stimulation, which is likely to be the cause of the increased basal proliferation of MZ B cells in αv-CD19 mice. The increased and prolonged activation of NF-κB in αv-deficient cells correlated with the extended association of TLR9 with early endosomes, suggesting that NF-κB signalling occurred from this compartment, in agreement with previous reports in myeloid cells^36^. MAP kinase (MAPK) signalling is thought to occur from the same compartment as NF-κB, and supporting this, levels of phospho-ERK and phospho-MAPK were increased at their peak and at later time points in αv-deficient cells compared with controls (Supplementary Fig. 6).

IRF7 activation in control B cells occurred later than NF-κB, at around 45 min, and reduced to background levels by 2 h (Fig. 5h). This is consistent with other studies showing that IRF7 signalling is triggered from a distinct endosomal compartment, and in our B-cell system this corresponded with the initial co-localization of TLR9 and LC3 in small endosome-like structures. In αv-deficient cells, IRF7 activation was significantly delayed, occurring at 2 h after TLR stimulation, suggesting to us that αv regulates transition of TLR9 from NF-κB-signalling compartments to endo-lysosomal compartments competent for IRF7 signalling, and that this may be mediated by recruitment of LC3. Notably this role for αv in LC3 re-organization and TLR signalling was not restricted to the TLRs that reside in endosomes, as αv-dependent LC3 re-organization was also seen in MZ and B-1 B cells after stimulation through TLR4 (Fig. 5i,j). Likewise, deletion of αv also led to prolonged NF-κB signalling downstream of TLR4 (Fig. 5k).

### αv is internalized to early endosomes during TLR signalling

**Figure 6:**
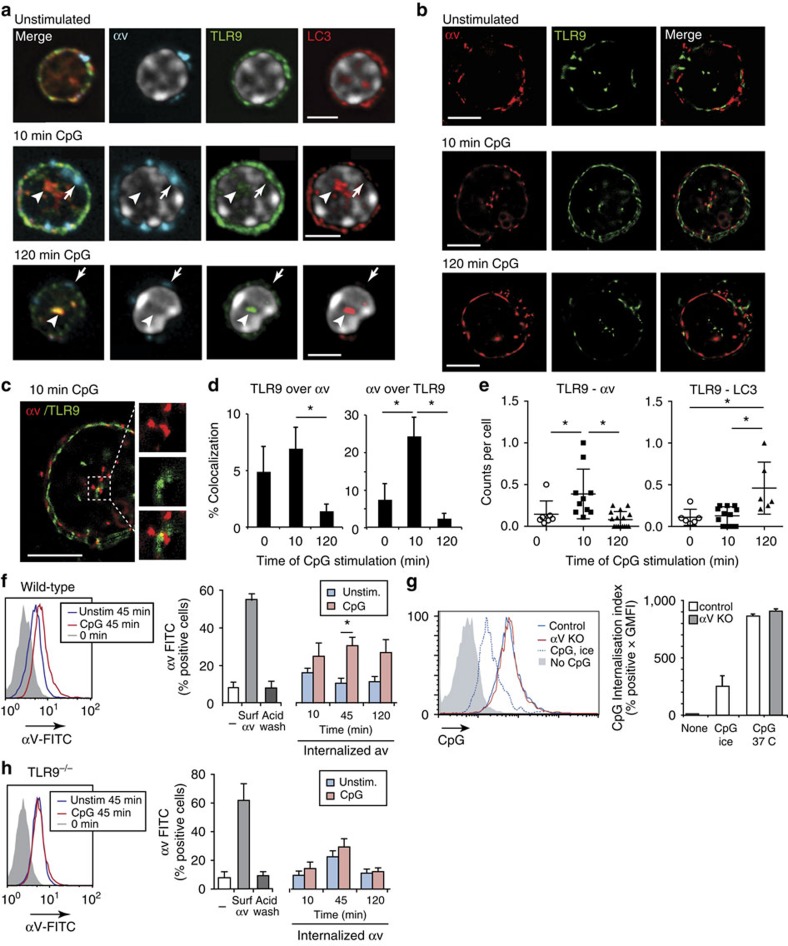
TLR9 stimulation leads to αv internalization and transient interaction of TLR9 and αv. (**a**) Peritoneal B-1 cells stimulated with CpG for indicated times, stained for TLR9, αv and LC3, and with Hoescht (omitted from merged images for clarity), analysed by confocal microscopy. Arrows indicate areas of TLR9 and αv co-localization, arrowheads areas of TLR9 aggregation without αv. Scale bars, 2.90 μm. (**b**,**c**) STORM images of sorted peritoneal B-1 cells stimulated with CpG and stained for TLR9 and αv antibody. Representative images of TLR9 and αv staining at different time points (**b**) and single images at 10 min CpG stimulation with selected regions of intracellular TLR9/αv co-localization are shown (**c**); scale bars, 2.50 μm. (**d**) Quantification of co-localization of αv and TLR9 over at least 30 cells imaged by STORM. (**e**) Association between TLR9 and αv or LC3 measured by proximity ligation assay and quantified as number of regions of positive co-localization per cell. Data are from analysis of at least 20 cells per condition. Similar results were seen in three independent experiments. Data in **d** and **e** are mean±s.d. *P<0.05, Student's *t*-test. (**f**) FACS plots and quantification of αv integrin internalization using anti-αv-FITC antibody in sorted MZ B cells from wild-type mice. Histograms show internalized αv-FITC at 45 min with or without CpG treatment. Bar graph shows % positive staining cells for αv-FITC in cells stained without washing (surface αv or surf), after acid wash, or without staining (−) as controls, as well as cells allowed to internalize αv-FITC for indicated times with or without CpG stimulation, followed by acid wash, permeabilization and neutralization. Experimental details are outlined in Supplementary Fig. 9. Bar graphs are mean±s.e.m. from four independent experiments. *P<0.01 Student's *t*-test. (**g**) Histogram plots show CpG internalization by control cells stimulated with CpG-alexa647 on ice and cells from control and αv-CD19 mice stimulated with CpG-alexa647 at 37 degrees. Cells were washed with cold acid wash buffer after stimulation to remove surface CpG staining and allow measurement of internalized material. Bar graph represents quantification of CpG internalization (mean±s.d. from one experiment, *n*=3 replicates; similar results were seen in 3 independent experiments). (**h**) FACS plots and quantification of internalized αv-FITC antibody in sorted MZ B cells from TLR9 knockout mice, measured as in **f**.

To understand the mechanism by which αv-directed traffic of TLRs, we followed localization of this integrin during stimulation of B cells. Visualizing αv by confocal microscopy, we observed some co-localization with TLR9, which was most pronounced in cells stimulated with CpG for 10 min (Fig. 6a). However, αv did not localize to the TLR9-LC3-positive lysosomal compartments seen 2 h after stimulation (Fig. 6a). To gain increased resolution of αv and TLR9 localization in these early time points after TLR stimulation, we used super-resolution microscopy (STORM)^45^. When isolated B-1 B cells were imaged in a single confocal section though the centre of the cell, we observed that αv and TLR9 co-localized in structures within the cell (Fig. 6b,c). The greater resolution available with STORM allowed us to more accurately quantify the degree of co-localization inside the cell, and revealed that this was highest shortly (10 min) after stimulation with CpG, declining to baseline levels by 2 h (Fig. 6c,d). To control for possible misalignment of fluorescent channels, during dSTORM imaging we used fluorescent beads as fiducial markers to align images for co-localization. However, the larger depth of focus used for wide-field dSTORM compared with confocal imaging meant that false signals could arise from proteins separated in the Z-plane; to exclude this possibility, we also used an independent measure of co-localization, proximity ligation assay (PLA)^46^. Confirming results from confocal and STORM microscopy, PLA showed αv and TLR9 co-localization was increased 10 min after CpG stimulation, but returned to control levels by 2 h (Fig. 6e; Supplementary Fig. 8). Also in agreement with our microscopy analysis, we observed significant TLR9-LC3 co-localization after 2 h (Fig. 6e). Together, these complementary microscopy and biochemical approaches demonstrate that αv co-localizes with TLR9 transiently, early after stimulation.

STORM imaging and PLA analysis suggested that αv-TLR9 co-localization occurs inside the cell, most likely in endosomes where TLR9 resides. To determine whether this involved αv internalization, we labelled cell surface integrin with fluorescently tagged antibody, incubated with CpG DNA, and then measuring internalized fluorescence. Using this technique, we found that CpG stimulated αv internalization, and that internalized αv was delivered to an acidic vacuole (Fig. 6f; Supplementary Fig. 9). Although αv integrins bind and mediate uptake of a range of complex self and microbial ligands, and can function as co-receptors for TLRs, it did not appear that αv was serving as an internalization receptor in this situation, as uptake of fluorescently labelled CpG DNA was unaffected by αv deletion (Fig. 6g). On the contrary, we observed that internalization of αv was dependent on TLR signalling, and decreased to background levels in TLR9 knockout B cells (Fig. 6h). We therefore concluded that TLR signalling promotes internalization of αv to early endosomal compartments where it co-localizes with TLR9.

### αv Promotes LC3 lipidation through Src-family kinases

**Figure 7:**
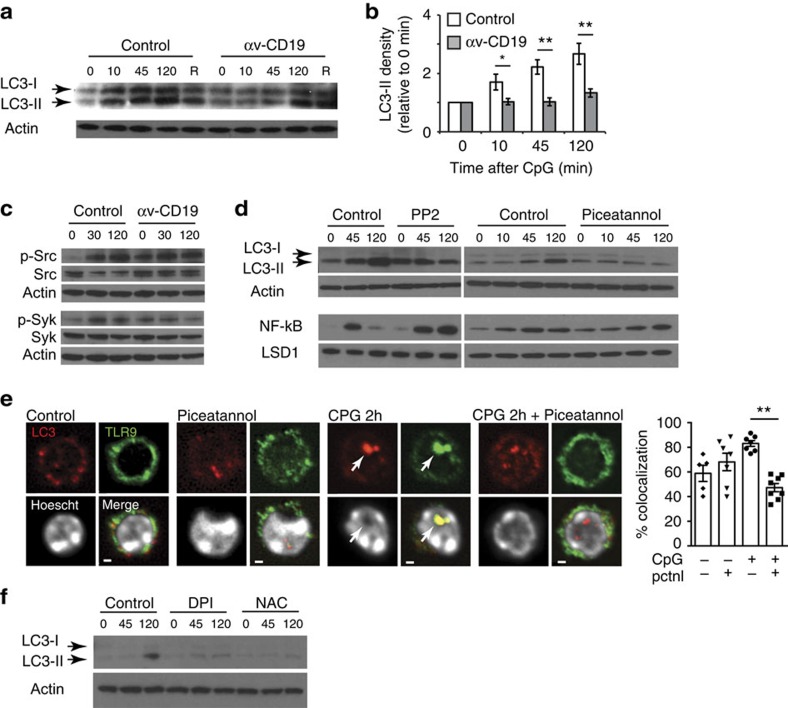
αv-mediated LC3 lipidation occurs through Src-family kinases. (**a**) Western blot of LC3 in sorted MZ B cells from control and αv-CD19 mice after stimulation with CpG DNA for 0–120 min, or stimulated with rapamycin (R) to induce autophagy. LC3-I and LC3-II forms indicated by arrows. Also shown is western blot of actin to normalize for protein loading. (**b**) Combined analyses of LC3-II level from four independent experiments. LC3-II quantified by densitometry was corrected for protein loading by actin, then expressed relative to levels at time 0, to allow combination of data from different experiments. Data show mean±s.e.m. *P<0.05, **P<0.01, Student's *t*-test. (**c**) Western blot of phospho-Src (Tyr 416) and phospho-syk (Tyr 525/526) kinases in sorted MZ B cells from control and αv-CD19 mice after CpG stimulation. Also shown are blots stained for total Src/syk, and actin. (**d**) Western blot of cytoplasmic LC3 and nuclear NF-κB in sorted MZ cells from control mice pre-treated with syk inhibitor (piceatannol) and Src inhibitor (PP2) as indicated, followed by CpG stimulation. Actin and LSD1 are shown as controls for total cellular and nuclear protein respectively. (**e**) MZ B cells treated with piceattanol and/or CpG for 2 h, stained for LC3 and TLR9, and analysed by confocal microscopy. Co-localization calculated by Pearson's coefficient, and displayed for individual fields of view, and mean±s.d. Data are combined from three independent experiments. **P<0.01 Mann–Whitney–Wilcoxon test. Scale bars, 0.70 μm. (**f**) Western blot of LC3 in sorted MZ cells from control mice pre-treated with two different ROS inhibitors DPI or NAC as indicated, before CPG stimulation. Arrows show position of LC3-II. Actin is shown as a control for protein loading. Full western blots are shown in Supplementary Fig. 10 and additional quantification of western blot analysis is in Supplementary Fig. 11.

Although αv localized with TLR9 early after stimulation, αv did not appear to mobilize with TLR9 into the LC3-positive compartments seen at 120 min (Fig. 6a), raising the question of how αv regulated the co-localization of TLR9 with LC3. Recruitment of LC3 to membranes requires conversion of cytosolic LC3 (LC3-I) to a lipidated form (LC3-II). TLR signalling induced LC3 conversion in MZ B cells, and this correlated in time with TLR9-LC3 co-localization (Fig. 7a,b). In αv-deficient B cells, LC3 lipidation was reduced and occurred much later than in control cells (Fig. 7a,b). This reduced LC3 conversion was not due to a fundamental defect in LC3 processing machinery in αv-knockout cells as processing through the macroautophagy pathway, induced by rapamycin, was unaffected by loss of αv, suggesting αv is required specifically for LC3 lipidation induced by TLR9.

Src-family protein tyrosine kinases, in particular Syk, have been implicated in lipidation and recruitment of LC3 to phagosomes^47^,^48^. Supporting a similar role for these kinases in TLR-triggered LC3 lipidation, we found that both Src and Syk were phosphorylated at activatory tyrosine residues following CpG stimulation of B cells (Fig. 7c). Src and Syk are both known to mediate intracellular signalling downstream of αvβ3 (ref. 49) suggesting this may be the mechanism by which αv couples TLR signalling to LC3 lipidation. To test this, we first assessed Src and Syk phosphorylation in αv-knockout cells. Confounding our experiments, basal phosphorylation of both kinases was higher in unstimulated αv-knockout MZ B cells than in control cells (Fig. 7c). Similar increased basal activation of Src-family kinases has been reported in numerous studies in which integrin signalling is inhibited^50^, and appears to be due to disruption of cell adhesion. However, of relevance to this study, stimulation of αv-knockout B cells with TLR ligands caused no increase in Syk phosphorylation and little, if any, increase in Src phosphorylation over levels in unstimulated cells (Fig. 7c; Supplementary Fig. 11). These data allowed us to place Syk activation downstream of αv, and suggested that Src activation is partially dependent on αv, consistent with existing models for integrin signalling in which activated Src binding to the β3 cytoplasmic tail amplifies Src phosphorylation and triggers activation of Syk^51^,^52^,^53^.

To test whether Src and Syk activation are required for LC3 conversion and co-localization with TLR9, we treated B cells with the pan Src-family kinase inhibitor PP2, or the specific Syk inhibitor, piceatannol. Neither inhibitor affected LC3-II generation in response to rapamycin, but both blocked conversion of LC3 in B cells treated with CpG DNA and led to prolonged nuclear localization of NF-κB (Fig. 7d; Supplementary Fig. 11), similar to the results seen in αv-deficient cells. Furthermore, Syk inhibition prevented co-localization of LC3 with TLR9 and the formation of large TLR9-containing vacuoles after CpG treatment (Fig. 7e). In phagocytes, Syk activates production of reactive oxygen species (ROS), which is required for recruitment of LC3 to phagosomes. In MZ B cells, inhibition of ROS with diphenyleneiodonium (DPI) or the anti-oxidant *N*-acetyl-L-cysteine (NAC) inhibited TLR9-induced LC3 lipidation (Fig. 7f), suggesting that Syk may work through similar mechanisms to recruit LC3 to endosomes. Taken together, these data demonstrate that αv directs co-localization of TLR9 and LC3 by facilitating the lipidation of LC3 through a mechanism involving Syk and Src-family kinases.

### Loss of LC3 or atg5 increase B-cell TLR signalling

**Figure 8:**
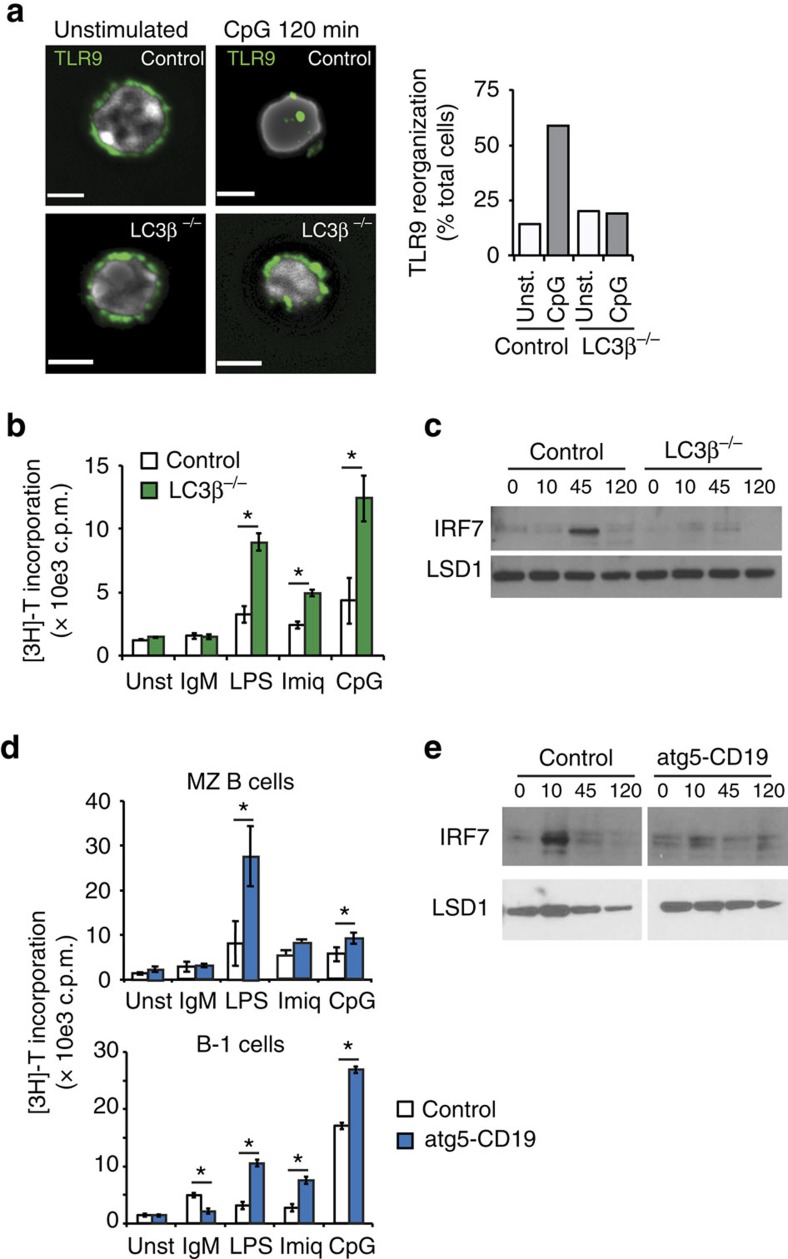
LC3 and atg5 are required to limit TLR signalling and activate IRF7. (**a**) MZ B cells from control and LC3β^−/−^ mice, untreated or cultured with CpG, stained for TLT9 and imaged by confocal microscopy. Hoechst staining (white) indicates the cell nucleus. Scale bar, 2.90 μm. Bar graph represents quantification of the cells with TLR9 re-organization, based on analysis of at least 60 cells per condition in two independent experiments. (**b**) Proliferation of sorted MZ B cells from LC3β^−/−^ mice in response to TLR ligands and anti-IgM. Each point is mean±s.d. of *n*=3 replicates. Similar results were seen in three independent experiments. (**c**) Western blot analysis of nuclear IRF7 in sorted MZ B cells from LC3β^−/−^ mice. LSD1 staining is included as protein loading control. (**d**) Proliferation of sorted MZ or sorted peritoneal B1 cells from atg5-CD19 mice stimulated with TLR ligands and anti-IgM. Each point is mean±s.d. of *n*=3 replicates. Similar results were seen in three independent experiments. (**e**) Western blot analysis of activation of IRF7 in nuclear extracts from MZ B cells sorted from atg5-CD19 mice.. LSD1 staining is included as protein loading control. *Significantly different *P*<0.05, Student's *t*-test. Full western blots are shown in Supplementary Fig. 12. Unst, unstimulated.

To determine whether LC3 recruitment is required for the regulation of TLR signalling by αv, we analysed responses of B cells from mice deficient in one member of the LC3/atg8 family, LC3β. In LC3β^−/−^ B cells, TLR9 did not re-localize to large vacuolar structures following CpG treatment (Fig. 8a), indicating that LC3 itself is required for this aggregation of TLR9. MZ B cells from LC3β^−/−^ mice had increased proliferative responses to TLR ligands, as we saw for αv-deficient cells (Fig. 8b). However, unlike our results with αv-CD19 mice, we saw no activation of IRF7 in LC3β^−/−^ B cells (Fig. 8c). To confirm that these effects were due to loss of LC3 lipidation machinery, we also assessed B cells from mice with B-cell-specific deletion of the autophagy regulator *Atg5* (Atg5-CD19 mice), which functions upstream of LC3 (ref. 54). For B-cell subsets that are not regulated by αv, such as freshly isolated peritoneal B-2 B cells, atg5-deficient cells showed reduced proliferation compared with control cells (Supplementary Fig. 13), consistent with previously reported roles for atg5 in B-cell survival^55^,^56^,^57^, which are thought to be due to loss of macroautophagy. Proliferation of B-1 B cells in response to BCR crosslinking, which is not regulated by αv, was also impaired in atg5-CD19 mice (Fig. 8d), consistent with the reported defect in B-1 cell survival in these mice^55^. However, in agreement with our observations in LC3β-deficient cells, proliferation of atg5-deficient MZ and B-1 B cells in response to TLR stimulation was increased compared with controls (Fig. 8d). Activation of MAPK, IKK and NF-κB following TLR stimulation was also increased in atg5-deficient B cells (Supplementary Fig. 13) whereas, nuclear localization of IRF7 was completely blocked (Fig. 8e). These data therefore confirm that atg5 and LC3β regulate TLR signalling through NF-κB and MAPKs, and affect B-cell activation, in similar ways to αv integrins. They also show that LC3 recruitment to TLR endosomes is absolutely required for IRF7 signalling. Together, these findings strongly support a model in which αvβ3-mediated activation of regulation of TLR responses occurs through activation of autophagy components and their association with TLRs (Supplementary Fig. 14).

## Discussion

Our data describe a new mechanism of regulation of B-cell activation by TLR ligands, mediated by the integrin αvβ3. We show that αv promotes the trafficking and maturation of TLR-containing endosomes, and through this mechanism, reduces polyclonal activation of B cells through TLRs. In mice, loss of this mechanism by deletion of αv from B cells leads to greater production of natural antibodies and increased responses to TLR-ligand-associated antigens, but also promotes production of autoantibodies. We therefore propose that this mechanism serves to maintain the balance between protective immunity to microbes and potentially pathological responses to self.

Our data reveal a link between αvβ3 signalling and two components of the autophagy pathway, LC3 and atg5, and identify two key functions for these autophagy components in TLR signalling. First, we show that αv, LC3 and atg5 reduce cellular response to TLR stimulation, and in the absence of any of these genes, B-cell proliferation in response to TLR ligands is greatly increased. Second, we also show that LC3 recruitment to TLR-containing endosomes is required for IRF7 signalling in response to TLR ligands. Based on our microscopy and biochemical data, we propose that TLR9 transitions through three endolyosomal compartments in B cells during signalling. Initially, TLR9 engages CpG DNA in an early endosome, marked by EEA1 and TfR, and signals through NF-κB. TLR9 then transitions to an LC3-positive endosomal compartment, in a process triggered by αvβ3-mediated LC3 lipidation. It is likely that activation of IRF7 occurs from this LC3^+^ endosomal structure, as IRF7 activation coincides in time with the initial lipidation of LC3 and association with TLR9 while TLR9 remains distributed throughout the cell, at around 45 min in control cells. TLR9 subsequently traffics to a much larger vacuolar structure, which stains intensely for LC3. At this time (2 h after stimulation), signalling through both NF-κB and IRF7 has ceased, and this therefore appears to represent a terminal point in TLR signalling. As this structure also contains LAMP-2, which is critical for fusion of phagosomes and auto-phagosomes with lysosomes, we predict that LC3 and other autophagy components cause termination of signalling by recruitment of lysosomal or proteasomal machinery, similar to their function in classical or selective autophagy, although this remains to be demonstrated. In the absence of αv-mediated, LC3-directed trafficking, TLR9 spends longer in early endosomes, leading to prolonged NF-κB signalling and delayed IRF7 activation, and we conclude that this is the cause for the increased cell proliferation and antibody production that we see in αv-knockout B cells. Our observation that αv regulates LC3 localization, NF-κB signalling and cell proliferation in response to the TLR4 ligand LPS strongly support a similar role for αv in signalling by TLRs that engage their ligands at the cell surface. However, although previous studies have described how the early progression of TLR-containing endosomes can affect the nature of the signal, our data emphasize that the duration of TLR in any compartment is a determinant of the strength of signalling triggered therein and reveal a hitherto unappreciated function of autophagy components in terminating signalling.

The model that we propose based on our data is different from that originally proposed for TLR9 signalling in pDCs, where IRF7 signalling was thought to originate from early endosomes and NF-κB signalling required transition to the lysosome^34^,^35^. These original studies were based primarily on the use of different CpG DNA ligands, which preferentially localized to endosomal or lysosomal compartments. More recent studies focusing on responses following alteration of intracellular trafficking and endosomal sorting are more consistent with our results, namely that NF-κB signalling occurs from early endosomes and that these require further maturation to become competent for IRF7 signalling^36^,^58^. Notably, recent work identified LC3 as a critical component of phagosomes that can signal through IRF7, in direct agreement with our work^59^. In addition, our interpretation is consistent with the model originally proposed for TLR4 (refs 37, 38), in which signalling through NF-κB and IRF pathways occur sequentially and requires transition of the TLR from the surface to endo-lysosomal compartments. However, it remains to be demonstrated whether the pathway we described in B cells functions in other immune cells.

Despite their initial identification in the context of autophagy, it is now apparent LC3, atg5 and related components are involved in a much broader range of membrane trafficking and re-organization events in cells. Notably, these, along with atg7, are intimately involved in internalization of microbial and self-derived particles, a process termed ‘LC3-associated phagocytosis'^60^. The process of αv-regulated LC3 activation and involvement in endocytic trafficking that we have identified has several parallels with LC3-associated phagocytosis. First, they appear to use similar mechanisms as LC3 recruitment to phagosomes also requires Syk and ROS^47^,^48^. Furthermore, LC3 is required for TLR9 signalling through IRF signalling in both cases (Fig. 8)^59^. Our data significantly extend these roles for LC3 and autophagy components in TLR signalling, demonstrating that their involvement in vesicular traffic and TLR signalling is not limited to phagosomes, and identifying an important role in limiting TLR signalling. Furthermore, our finding that deletion of a single LC3/atg8 homologue, LC3b, disrupts this mechanism raise the possibility that specific components of this pathway are specialized for roles in immune signalling.

TLR9 recruitment to auto-phagosome-like compartments has previously been reported in B cells^41^. In that study, engagement of the BCR was required to drive TLR recruitment. In contrast, our data suggest that TLR trafficking occurs independently of the BCR and instead requires αv. Although seemingly at odds with our observations, an important difference in the subset of B cells studied must be considered. The previous study of Chaturvedi *et al*. used total spleen B cells, the majority of which will be un-activated follicular B cells; in contrast, we show that the αv-mediated mechanism occurs only in MZ, B-1 and activated B cells. Curiously, the Syk-dependent pathway of integrin signalling implicated in our study shares many components with BCR signalling^53^ and it is interesting to speculate that following activation, αvβ3 may functionally substitute for the BCR in regulating TLR localization and signalling in B cells. Our data show that this switch to αv-mediated regulation in activated B cells is associated with increased surface expression of αvβ3; however it is possible that additional changes in signalling components must also occur to enable this mechanism, such as immunoreceptor tyrosine-based activation motif (ITAM)-containing adaptors that can link αvβ3 to Syk signalling^49^,^53^, and have been implicated in inhibition of TLR signalling. In other cell systems, Syk interaction requires αvβ3 ligand occupancy^49^, and whether specific ligand engagement and/or activation of αvβ3 are also required for integrin-mediated Src and Syk activation in activated B cells remains to be determined. Furthermore, it will be interesting to determine how closely this mechanism of αvβ3-mediated inhibition of TLR signalling relates to that reported for integrin αMβ2 (ref. 61).

Our findings support an emerging paradigm in which integrins affect diverse cellular processes by directing endosomal trafficking^39^,^40^. Of particular relevance to our results, αvβ3 has been shown to regulate growth factor signalling by altering the trafficking of receptors between recycling endosomes, where they can continue to signal, or the lysosome, where they are degraded. We find that αv plays an analogous role is directing the movement of TLRs from early endosomes to LC3-associated compartments, and that this is associated both with a transition from NF-κB to IRF7 activation and with termination of signalling. Therefore, we propose that although in some settings αv can mediate internalization of ligands, a more important role is in directing subsequent intracellular trafficking events. These findings explain how αv-mediated phagocytosis promotes trafficking of internalized material for degradation and antigen presentation in previous studies^62^,^63^. Intriguingly, in both of these cases other receptors could compensate for loss of αv to mediate phagocytic uptake, whereas the role of αv in directing the intracellular fate of phagocytic cargo was non-redundant. Taken together with our data, it therefore becomes possible to formally dissociate receptors involved solely in internalization of ligands from those such as αvβ3 that also regulate intracellular trafficking.

In summary, our data reveal a novel role for αvβ3 and atg5/LC3 in regulating TLR signalling in B cells. Polymorphisms in atg5 have recently been linked with SLE^64^,^65^, raising the possibility that disruption of the regulatory mechanism we describe may contribute to autoimmunity in human disease. In support of this idea, we find that loss of this regulatory mechanism leads to activation of potentially autoreactive MZ and B1 cells and increased production of antibodies to self-antigens, although we do not see evidence that αv-CD19 mice develop full autoimmunity. We therefore propose that this mechanism of αvβ3-regulated, LC3-directed trafficking helps to maintain the balance between rapid production of broad specificity, low affinity antibodies from MZ and B-1 B cells early in infections while preventing potential development of high affinity autoantibodies. An important conclusion from this study is to identify integrin signalling in B cells as a previously unappreciated mechanism of immune regulation. Extrapolating from these findings, we suggest αvβ3 as a potential new target for therapeutic interventions—blocking αvβ3 signalling would be predicted to augment beneficial vaccine-induced responses whereas αvβ3 agonists might limit detrimental self-directed antibody production.

## Methods

### Mice

αv-CD19 mice were generated as previously described^10^ and maintained on a mixed C57Bl/6/ 129Ola background. Littermates with a single CD19-Cre allele and Itgav-flox alleles were used as controls. As the background strains of these mice differ at their Igh-1 loci, and in their ability to make IgG2a versus IgG2c^82^, for immunization experiments mice were matched by Igh-1 genotype (all were Igh-1 a/b). The following mouse strains have been previously described and were used in this study on the following genetic backgrounds: Atg5-CD19 (ref. 64), TLR9^−/−^ (ref. 66) and GFP-LC3 (ref. 67) (all C57BL/6), LC3B^−/−^ (ref. 68), Integrin β3^−/−^ (ref. 69) and β5^−/−^ (ref. 70; all mixed C57BL/6/129). All mice were housed under specific pathogen free conditions at Benaroya Research Institute, Massachusetts General Hospital, Massachusetts Institute of Technology and University College London.

Animal experiments were performed under licences from the Institutional Animal Care and Use Committees of Massachusetts Institute of Technology, Massachusetts General Hospital and Benaroya Research Institute, within local and national guidelines for animal care.

### Antibodies and reagents

Anti-mouse CD21-APC (7G6), anti-mouseCD23-PE (B3B4), anti-mouseB220-PE-Cy7(RA3-6B2), anti-mouseB220-APC(RA3-6B2), anti-mouseCD11b-PE(M1/70), anti-mouse IgM-PE(R6-60.2), anti-mouse IgD-FITC(11-26c.2a), anti-mouse CD2-FITC(RM2-5) anti-mouse CD19-APC(1D3), anti-mouse CD71(C2)-biotin, anti-integrin αv (RMV-7)-biotin and Mouse BD Fc block (2.4G2) were from BD bioscience. Anti-TLR9 (26C593.2) was from IMGENEX. Anti-Calnexin (ab22595), anti-EEA1 (ab50313) and anti-Lamp2a (ab18528) were from Abcam. Goat anti-rabbit IgG (H+L) Alexa Fluor 546, Streptavidin Alexa Fluor-647, antibody to Src phosphorylated at Tyr 418 and Annexin v alexa fluor 488 were from Invitrogen Molecular probes. Hoechst 33342 was from Immunochemistry. Type C -CpG-ODN 2395, GpC-control ODN 2395, Type B CpG ODN 1826 and Imiquimod were from Invivogen. Antibody to ERK phosphorylated at Thr202 and Tyr 204 (20G11); antibody to p38 phosphorylated at Thr180 and Tyr182 (12F8), antibody to Syk phosphorylated at Tyr (525/526) antibody to IKKα-IKKβ phosphorylated at Ser176 and Ser180 (16A6), anti-NFkappa-B (D14E12), anti-Syk, anti-Src, anti-LSD1 (C69G12) and horseradish peroxidase conjugated anti-rabbit IgG were from Cell Signaling Technology. Anti-MyD88 (3244-100) was from Biovision. Anti-LC3B, anti-β-actin (AC-15), propidium iodide, 5-Bromo-2′-deoxyuridine, LPS (from *E. coli* O55:B5), Rapamycin from *Streptomyces hygrsocopicus*, DPI chloride and NAC were from Sigma-Aldrich. Alkaline phosphatase-conjugated anti-mouse IgG-AP, alkaline phosphatase-conjugated anti-mouse IgM-AP and anti-mouse IgG(H+L) were from Southern Biotech. CFSE (5 and 6 carboxyfluorescein diacetate succinimidyl ester)-(5)(6)-CFDA-SE was from Molecular probes. Thymidine methyl [^3^H]-Thymidine was from Perkin Elmer. Affinity purified F(ab')2 fragment goat anti-mouse IgM μ chain specific was from Jackson Immuno Research. Anti-mouse atto-488 was from (Hypermol, EK, Germany).

### Flow cytometry and cell sorting

Cells were harvested in phosphate-buffered saline (PBS)/0.5% bovine serum albumin (BSA)/2 mM EDTA and splenocytes or bone marrow cells depleted of red blood cells (RBC lysis buffer, Sigma-Aldrich). Single cell suspensions were blocked with Fc Block (BD Biosciences) and stained with fluorochrome tagged antibodies for surface markers (1: 200 dilution) at 4 °C for 30 min. Samples were acquired using FACSCalibur (BD Biosciences) and analysed by FlowJo software (Tree Star Inc.). For sorting of spleen MZ and follicular cells, cells enriched by lympholyte gradient (LympholyteM, Cedar Lane) were labelled with anti-B220-PE-CY7, anti-CD23-PE and anti-CD21-APC antibodies then sorted with SORPVantageSE Diva (BD Bioscience). For sorting of peritoneal B-1 and B-2 cells, peritoneal cavity cells were labelled with anti-B220-APC, anti-C11b-PE antibodies and sorted as above. For CpG uptake assay, sorted MZ cells were rested and stimulated with 1 μM CpG Alexa Fluor-647 on ice or 37 °C for 30 min. Cells were then washed three times in ice-cold PBS followed by one wash in cold acid wash buffer (PBS with 0.2 M acetic acid and 0.5 M NaCl) for 5 min to remove CpG bound on surface, and analysed by FACS. For integrin internalization assay sorted MZ cells were labelled with integrin αv-FITC (Hmα5-1, EBioscience^4^) antibody on ice for 30 min and assayed as described in Supplementary Fig. 9. Surface bound antibody was then allowed to internalize for different times points with or without CpG stimulation at 37 °C in regular growth medium. At each time point cells were washed with cold PBS and remaining antibody at surface was removed by two acid washes for 2 min on ice. Cells were re-suspended in PBS/0.5% triton to neutralize intracellular vacuoles and recover quenched internalized fluorescence, and analysed by FACS.

### *In vitro* proliferation assays

Sorted cells were plated in X-VIVO15 (Lonza) supplemented with 2 mM glutamine, 100 U ml^−1^ penicillin and 100 μg ml^−1^ streptomycin, and 50 μM 2-β-mercaptoethanol or complete RPMI-1640 (10% fetal bovine serum 2 mM glutamine, 100 U ml^−1^ penicillin and 100 μg ml^−1^ streptomycin, and 50 μM 2-β-mercaptoethanol) at a density of 3 × 10^4^ cells per well on 96-well plate and treated with different stimuli: LPS (1 μg ml^−1^), CpG or control GpC (1.5 or 3 μM), Imiquimod (10 μM), anti-IgM F(ab′)2 (10 μg ml^−1^) and anti-CD40 (10 μg ml^−1^). After 48 h cells were pulsed with 1 μCi/well [3H]-Thymidine for 18 h before harvest; incorporation was determined by liquid scintillation spectrometry. To measure proliferation by CFSE labelling, sorted cells were stained for 5 min at room temperature with 1 μM CFSE, washed with PBS/5% fetal bovine serum and plated as above. For proliferation of activated follicular cells, FACS-sorted follicular cells were cultured overnight with or without anti-IgM stimulation (5 μg ml^−1^) and washed extensively before stimulation with TLR ligands as mentioned above.

### *In vivo* proliferation

Mice were injected with 100 μg CpG-ODN intraperitoneally (for analysis of peritoneal cavity cells) or intravenously (for spleen cells), fed with bromodeoxyuridine (BrdU) continuously via the drinking water (0.8 mg ml^−1^ BrdU Sigma-Aldrich, and 10% sucrose) and killed on day 4. Additional mice were given BrdU water for the same time without CpG injection. Cells from spleen and peritoneal cavity were surface stained, then fixed and permeabilized, treated with DNase, and stained with anti-BrdU FITC (BD Biosciences).

### Confocal microscopy

FACS-sorted MZ B cells, or enriched peritoneal B cells were seeded on to poly-L-lysine coated glass cover slips (BD Bio-coat) in complete RPMI-1640 and allowed to attach for 1 h at 37 °C. Next, cells were stimulated with 3 μM CpG for the indicated times. After stimulation cells were fixed with 4% PFA for 20 min at room temperature, permeabilized with saponin (0.2% saponin in PBS, 0.03 M sucrose, 1% BSA) for 10 min at room temperature. Non-specific binding in cells was prevented by incubating for 1 h at room temperature with blocking buffer (2% goat serum, 1% BSA, 0.1% cold fish skin gelatin, 0.1% saponin, 0.05% Tween-20 in 0.01 M PBS, pH 7.2). Cells were incubated overnight at 4 °C with specific primary antibodies (TLR9 1:250 dilution, LC3 1:500 dilution, EEA1 and TfR 1:500 dilution) in dilution buffer (PBS, 0.05% Tween-20, 1% BSA, 0.1% saponin). Cells were washed three times in dilution buffer (5 min each) and incubated in secondary antibodies (1: 500 dilution) in dilution buffer for 1 h at room temperature. Next, cells were washed three times in dilution buffer and twice in PBS. Cell nuclei were stained with Hoechst 33342, and the cover slips were mounted with ProLong Antifade (Invitrogen). Cells were imaged through a 100 × oil objective (aperture 1.4) and Nikon Ti (Eclipse) inverted microscope with Ultraview Spinning Disc (CSU-X1) confocal scanner (Perkin Elmer). Images were captured with an Orca-ER Camera using Volocity (Perkin Elmer). Post acquisition analysis such as contrast adjustment, deconvolution through iterative restoration and three-dimensional reconstruction were performed using Volocity software. For quantitative analysis of the co-localization of TLR9 and LC3 or endosomal markers (EEA1 and TFR) Pearson's correlation was calculated using Volocity software. Proximity ligation assay was performed using the Duolink In Situ Red Starter Kit Mouse/Rabbit from Sigma-Aldrich. Peritoneal B cells were cultured on poly-L-lysine coated coverslips and stimulated and stained as above for confocal microscopy. The primary antibodies used were anti-TLR9, anti-αv and anti-LC3 and PLA probes anti-rabbit PLUS and anti-mouse MINUS were used with detection reagent red according to Duolink protocol. Cells were imaged using confocal microscope as above and ImageJ was used to quantify PLA signal. For statistical analysis the number of positive foci were counted in >25 total cells in multiple (>10) fields of view per condition.

### STORM imaging

Peritoneal B cells or sorted B1 cells were seeded on to poly-L-lysine coated chamber slides (Nunc Lab-Tek, ThermoScientific) stimulated, fixed and stained as for confocal microscopy using anti-mouse atto-488 (Hypermol, Bielefeld, Germany) and streptavidin 647 as secondary reagents. Samples were imaged at room temperature on a dSTORM Diskovery imaging system (Molecular Devices, Sunnyvale, CA, USA), which consisted of a Leica DMI 6000B microscope, with 100 × objective and a ‘Perfect Focus' system. Wide-field imaging was performed in an extracellular solution containing reducing and oxygen scavengers, as specified by dSTORM protocols. The fluorochromes were first converted to a dark state using a 637 nm laser at 140 kW, 561 nm at 150 mW, or 488 nm at 150 mW (Spectral Applied Research, Richmond Hill, Ontario, Canada). Once the fluorochromes were converted into a desired density of single molecules per frame, the laser power was reduced and cells were imaged continuously at 8,000–10,000 frames. Tetraspeck beads 100 nm (with four, well-separated excitation/emission peaks: 360/430 nm (blue), 505/515 nm (green), 560/580 nm (orange) and 660/680 nm (dark red)) (Invitrogen) were used as fiducial markers to align images. Beads were adsorbed on the coverslip with the cells, and were in the same field of view as cells during image acquisition. The microscope stand (LEICA DMI6000) was equipped with hardware autofocusing and continuous focusing, which kept the sample in focus and at least one bead was in the field of view during image acquisition for each fluorescence channel. dSTORM images were captured at a focal point midway through the cell. Localization was acquired using a Photometrics Evolve 512 × 512 backthinned EM-CCD with 16 micron pixels and analysed with MetaMorph Imaging Software, MetaSeries version 7.8.2 (Molecular Devices, Sunnyvale, CA, USA) using a 10 ms exposure time. Images were reconstructed and analysed in real-time using the integrated WaveTracer. The WaveTracer module was used to determine co-localization by examining overlapping pixel signals from each fluorescence channel in the reconstructed image. Co-localization analysis was restricted to intracellular regions of the cell (determined by exclusion of the ‘rim' of strong αv staining which marks the plasma membrane).

### ELISA

Immulon 2HB microtitre plates (DYNEX) were coated and dried overnight at 37 °C with salmon sperm DNA (100 μg ml^−1^ in TE buffer). DNA was nitrocellulose filtered for dsDNA or heated for 5 min at 100 °C for ssDNA. After blocking for 60 min at 37 °C (2% BSA, 2% fetal calf serum, 0.1% Tween-20 and 0.02% sodium azide in PBS) sera diluted 1:100 in 50% block were applied (60–120 min at 37 °C) followed by alkaline phosphatase-conjugated goat anti-mouse IgM or IgG (Southern Biotech) diluted in blocking buffer for 60 min at 37 °C. Secondary antibodies were detected by using disodium p-nitrophenyl phosphate substrate (Sigma-Aldrich) and absorbance (OD) read at 405 nm. For detection of total Ig in culture supernatants or sera plates were coated with 10 μg/ml goat anti-mouse IgG (heavy and light chain specific; Southern biotech) in PBS at 4 °C overnight. Plates were blocked with 1% BSA in PBS and incubated with various dilutions of serum sample or cell culture supernatants in PBS. After incubation with 1:1,000 diluted alkaline phosphatase-conjugated IgM, IgG, IgG1 or IgG2c (Southern Biotech) colour was developed as above with disodium p-nitrophenyl phosphate substrate.

### Western blots

Cells were lysed for 30 min on ice in RIPA buffer containing 1% NP-40, 0.5% sodium deoxycholate, 0.1% SDS supplemented with sodium orthovanadate and protease inhibitor cocktail. Nuclear extracts were prepared by lysing cells in hypotonic nuclear extraction buffer (1 M Hepes, PH7.5, 5 M NaCl, 0.5 M EDTA PH8, 50% glycerol, 10% Igepal and 10% TritonX100) for 10 min followed by centrifugation at 1,500*g* for 5 min at 4 °C to pellet the nuclei and nuclei were re-suspended in RIPA buffer. Lysates were centrifuged for 10 min at 4 °C at 14,000*g* and supernatant was collected as nuclear fraction. Proteins were quantified by BCA assay (Pierce), separated by electrophoresis using NuPage-Bis-Tris gels (Invitrogen) and blotted onto PVDF membranes. Non-specific binding was blocked with 5% BSA in TBS-Tween (0.1%) followed by incubation with primary antibodies (1:1,000 dilution) overnight at 4 °C and secondary antibody horseradish peroxidase conjugated antibodies (1:2,000 dilution) for 1 h at room temperature. Membranes were washed thoroughly with TBS-Tween (0.1%) after antibody incubations and developed using ECL reagents (Millipore). For re-probing blots were stripped for 30 min at 37 °C with Restore PLUS stripping buffer (Thermo Scientific). For experiments with inhibitors cells were pre-treated with PP2 (1 μM), Piceatannol (25 μM), DPI (25 μM) and NAC (5 mM) for 30 min and cells were stimulated and processed in RIPA or nuclear lysis buffer as above.

### Immunizations

For T-independent immune responses, mice were immunized i.p. with NP-haptenated Ficoll in PBS at the doses indicated in figures. For experiments using TLR ligands and alum as adjuvants, mice were immunized i.p with NP-CG (Biosearch technologies) 50 μg per mouse in combination with alum (1:1 suspension) or LPS (5 μg per mouse).

## Additional information

**How to cite this article:** Acharya, M. *et al*. αv Integrins combine with LC3 and atg5 to regulate Toll-like receptor signalling in B cells. *Nat. Commun.* 7:10917 doi: 10.1038/ncomms10917 (2016).

## Supplementary Material

Supplementary Information Supplementary Figures 1-15
