# Supplementary material for: αv Integrins combine with LC3 and atg5 to regulate Toll-like receptor signalling in B cells
Source: Nat Commun. 2016 Mar 11;7:10917. doi: 10.1038/ncomms10917 (PMC4792966; doi:10.1038/ncomms10917)
Supplement: Supplementary Information — Supplementary Figures 1-15 [file ncomms10917-s1.pdf]

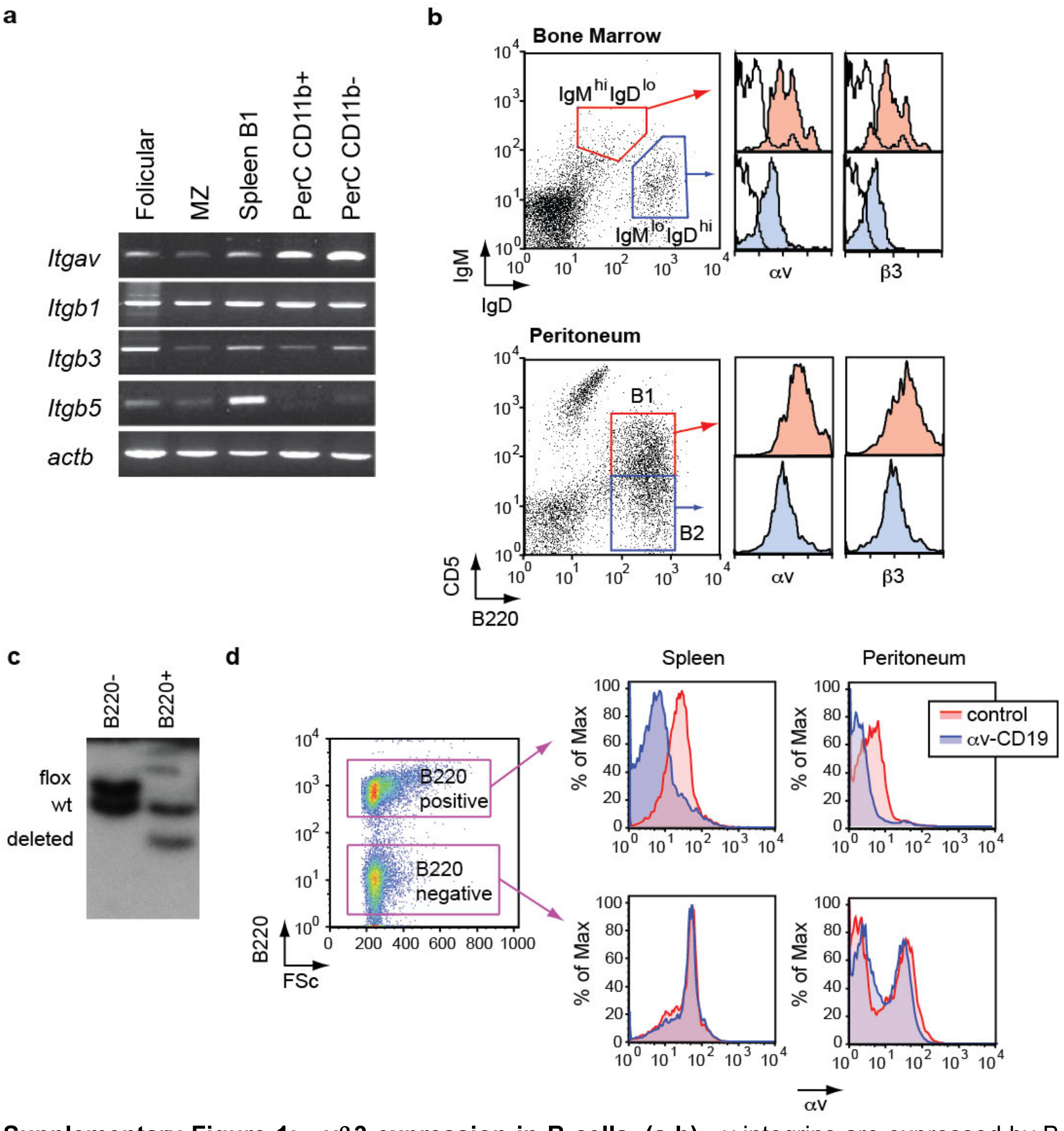

Supplementary Figure 1: αvβ3 expression in B cells. (a-b) αv integrins are expressed by B cells. **(a)** Expression of mRNA for αv and pairing β integrin mRNAs in sorted B cell subpopulations (Spleen follicular, MZ and B-1 B cells, peritoneal CD11b+ and CD11b- B cells [representing peritoneal B-1 and B-2 cells]). **(b)** Surface expression of αv and β3 integrins on bone marrow and peritoneal B cells. Histograms show staining for αv or β3 (filled histograms) and isotype control antibody (open histograms) on B cells as indicated. **(c-d)** Specific deletion of *Itgav* gene in B cells of αv-CD19 mice. **(c)** Genomic DNA from sorted spleen B220^{+} and B220^{-}

cells from *Itgav* $^{flox/+}$: *cd19-cre* mice was analyzed by southern blot to identify wild type, floxed and deleted alleles of the *Itgav* gene. The floxed allele was replaced by the deleted allele in $B220^+$ B cells. **(d)** FACS analysis of spleen and peritoneal B cells from αv-CD19 mice and controls, stained for B220 and αvβ3. Cells were gated on $B220^+$ and $B220^-$ cells as indicated for spleen cell staining; similar gating was used in peritoneal cells. αvβ3 staining was selectively lost in $B220^+$ cells from αv-CD19 mice (blue histograms) compared with controls (red histograms). Similar results were seen in 3 independent experiments.

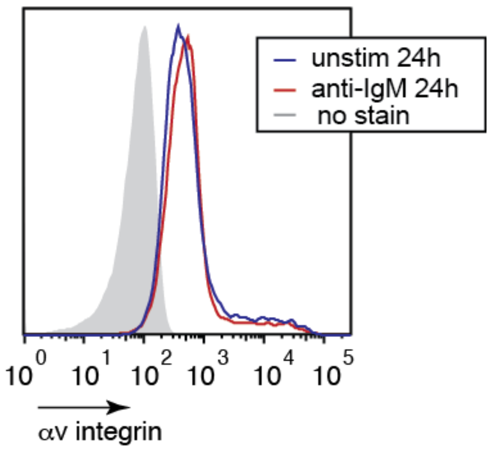

Supplementary Figure 2: αv expression on follicular B cells cultured overnight with anti-IgM antibody. FACS-sorted follicular B cells were cultured for 24 h without stimulation (unstim) or with anti-IgM Fab2 fragments (5μg/ml). Cells were then anlayzed for surface αv expression by FACS. Histogram plots show unstained cells (grey solid histogram) and expression of surface αv on cells with (red) or without (blue) IgM stimulation**.**

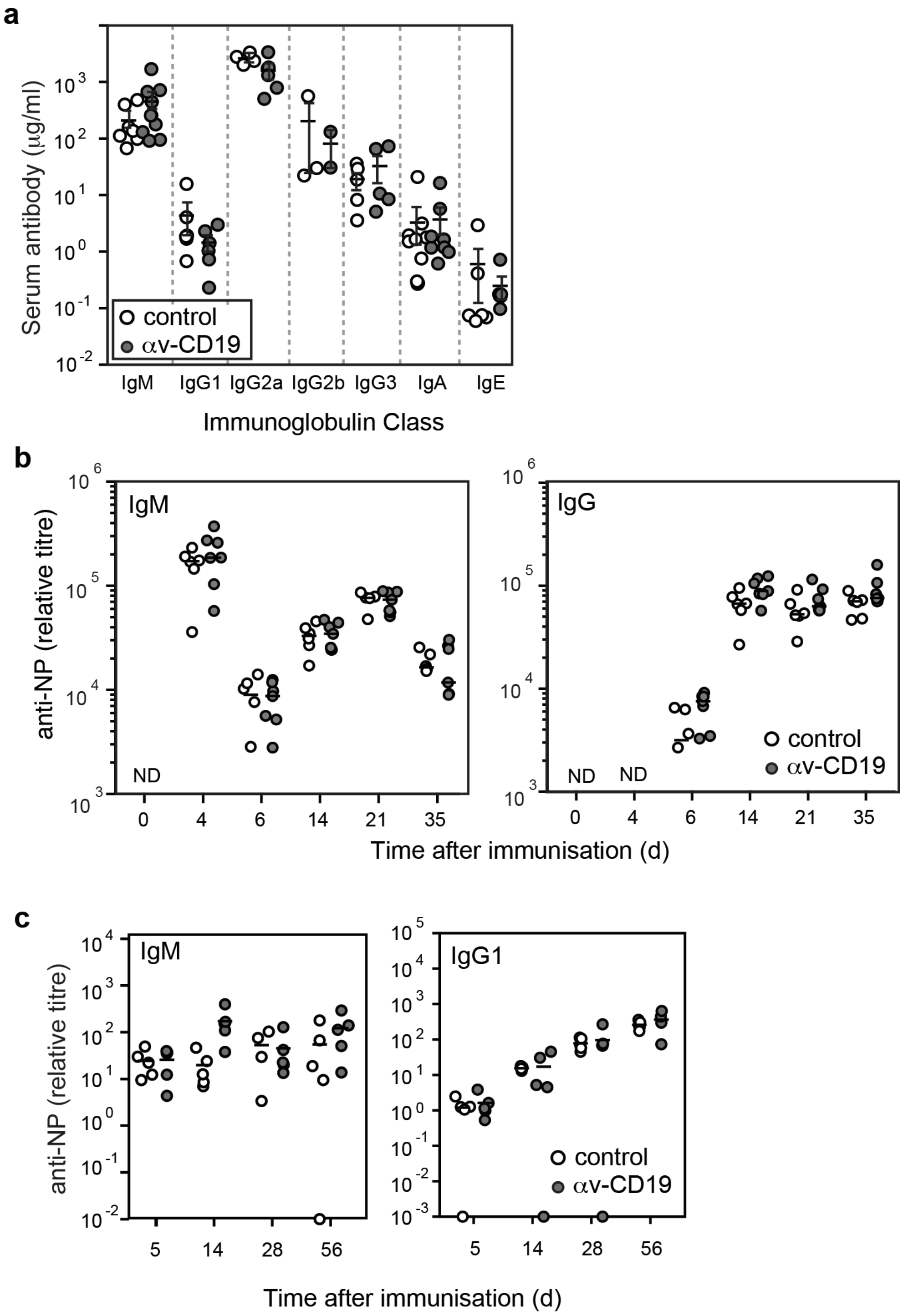

Supplementary Figure 3: Antibody responses in αv-CD19 mice. (a) Total serum immunoglobulin titres in non-immunized αv-CD19 and littermate control mice. **(b-c)** Serum anti-NP antibody titres of indicated class following immunization of αv-CD19 mice and littermate controls with NP-CG in LPS **(b)** or NP-CG in alum **(c)**, measured at 5-56 days after immunization. In all cases, each dot represents a single mouse, bars show mean.

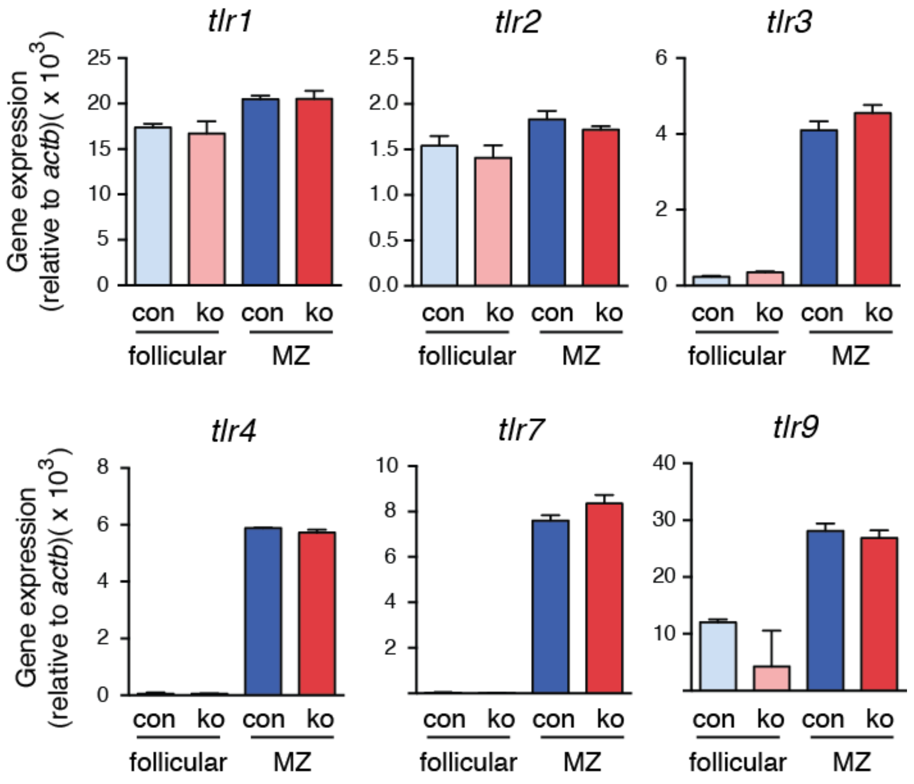

Supplementary Figure 4: TLR expression by αv-deficient B cells. Quantitative real-time PCR analysis for expression of various toll-like receptors (tlr) on RNA isolated from sorted Marginal Zone (MZ) or Follicular B cells from control mice (con) or αv-CD19 mice (ko). Bar graph represent gene expression relative to expression of housekeeping gene β-actin, and show mean ± sd of triplicate samples.

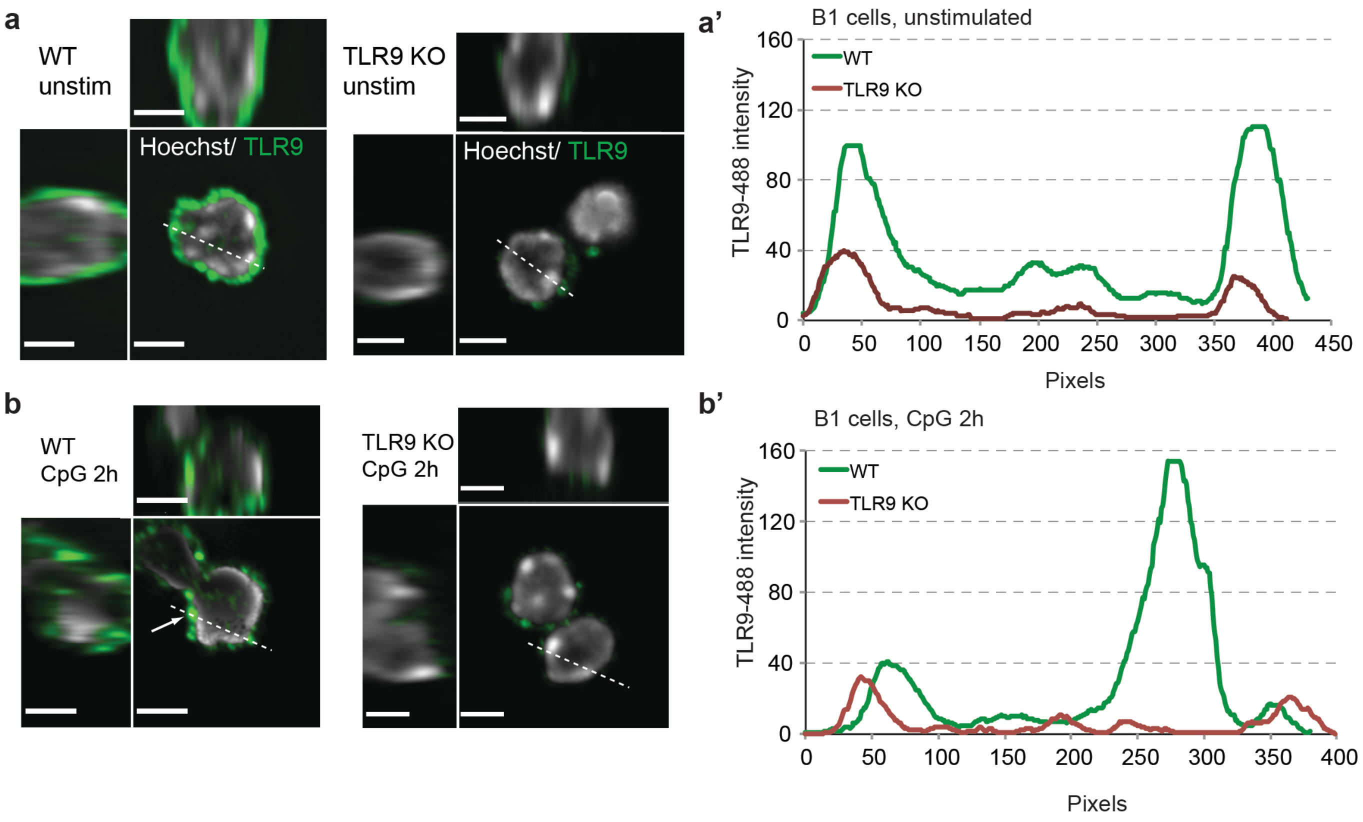

Supplementary Figure 5: TLR9 can be specifically detected by antibody (a-b) Peritoneal B-1 cells from wild-type (WT) and TLR9 knockout (KO) mice were stained with anti-TLR9 antibody either before or after 2 hours stimulation with CpG DNA, analyzed by con-focal microscopy. Images show TLR (green) and DNA (Hoescht stain, white) in xy , xz and yz planes. Histograms **(a', b')** show fluoresecence intensity across the cell (plane marked by dotted white lines in images). Note both WT and KO cells were stained together and imaged with the same fluorescence intensity and detection parameters. Scale bar, 2.90 μm

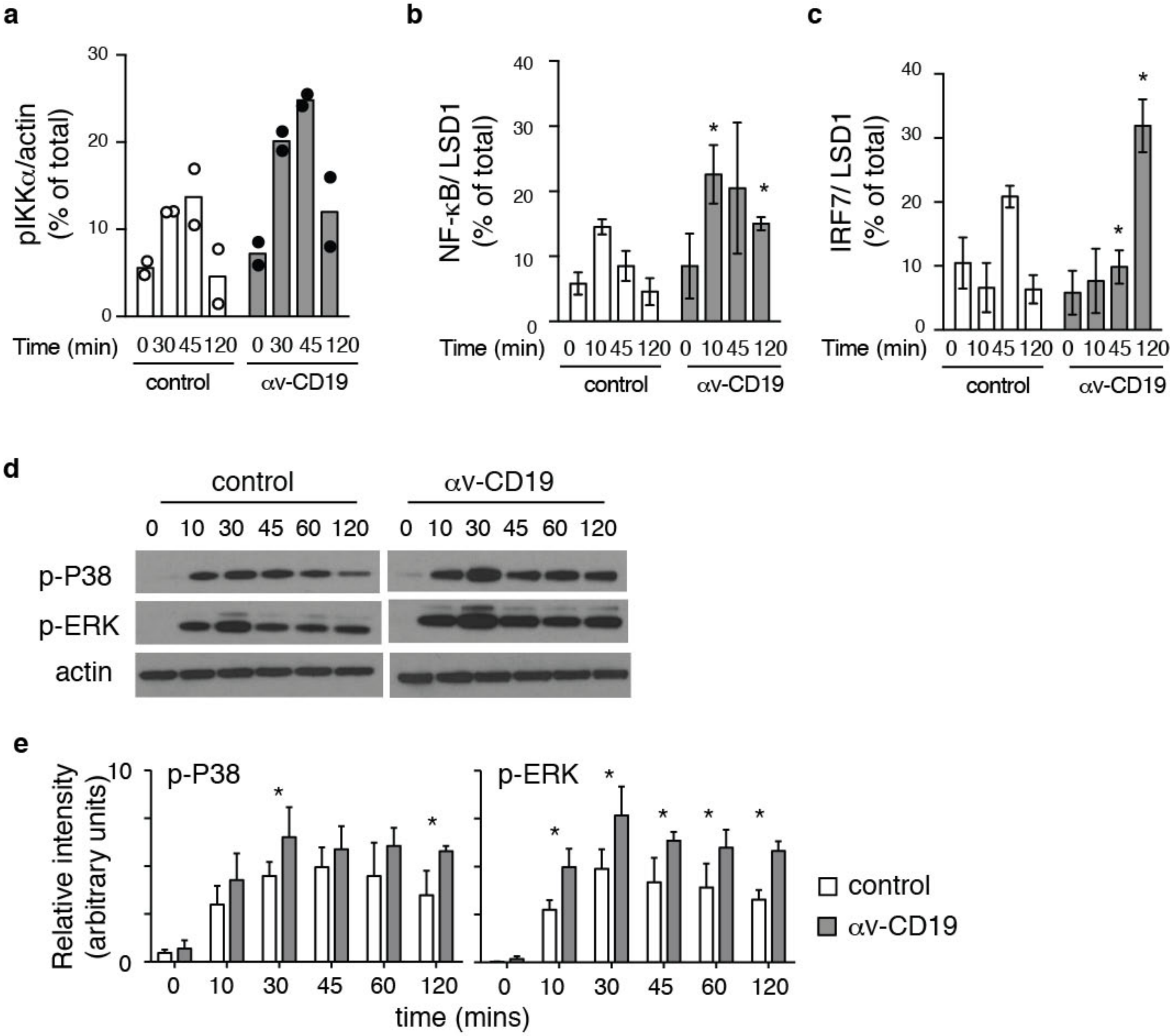

Supplementary Figure 6: Increased TLR signaling in αv-deficient B cells. (a-c) Quantification of western blots of cytoplasmic phospho-IKK (pIKK)**(a)** and nuclear NF-κB **(b)** and IRF7 **(c)** in sorted MZ B cells isolated from αv-CD19 and control mice, stimulated with CpG DNA for the indicated time (mins). In all cases, specific protein bands were first normalized to actin or LSD1 (for cytoplasmic and nuclear proteins respectively) to control for total protein recovery and loading between samples. Corrected protein levels were then expressed as a percentage of total signal on the western blot to allow comparison between experiments. Data shown are mean and individual values (pIKK) or mean ± sd (NF-κB, IRF7) for n ≥ 3 independent experiments, and analyses include western blots presented in Figure 5. *, significantly different from corresponding control, $p<0.05$, Student's t-test. **(d,e)** Representative Western blot **(d)** and quantification **(e)** of p-p38 and p-pERK in sorted MZ B cells isolated from αv-CD19 and control mice, stimulated with CpG DNA for the indicated time (mins). Also shown are staining of actin to confirm loading of similar amounts of protein. Quantification and statistic were as in **(a-**c) above.

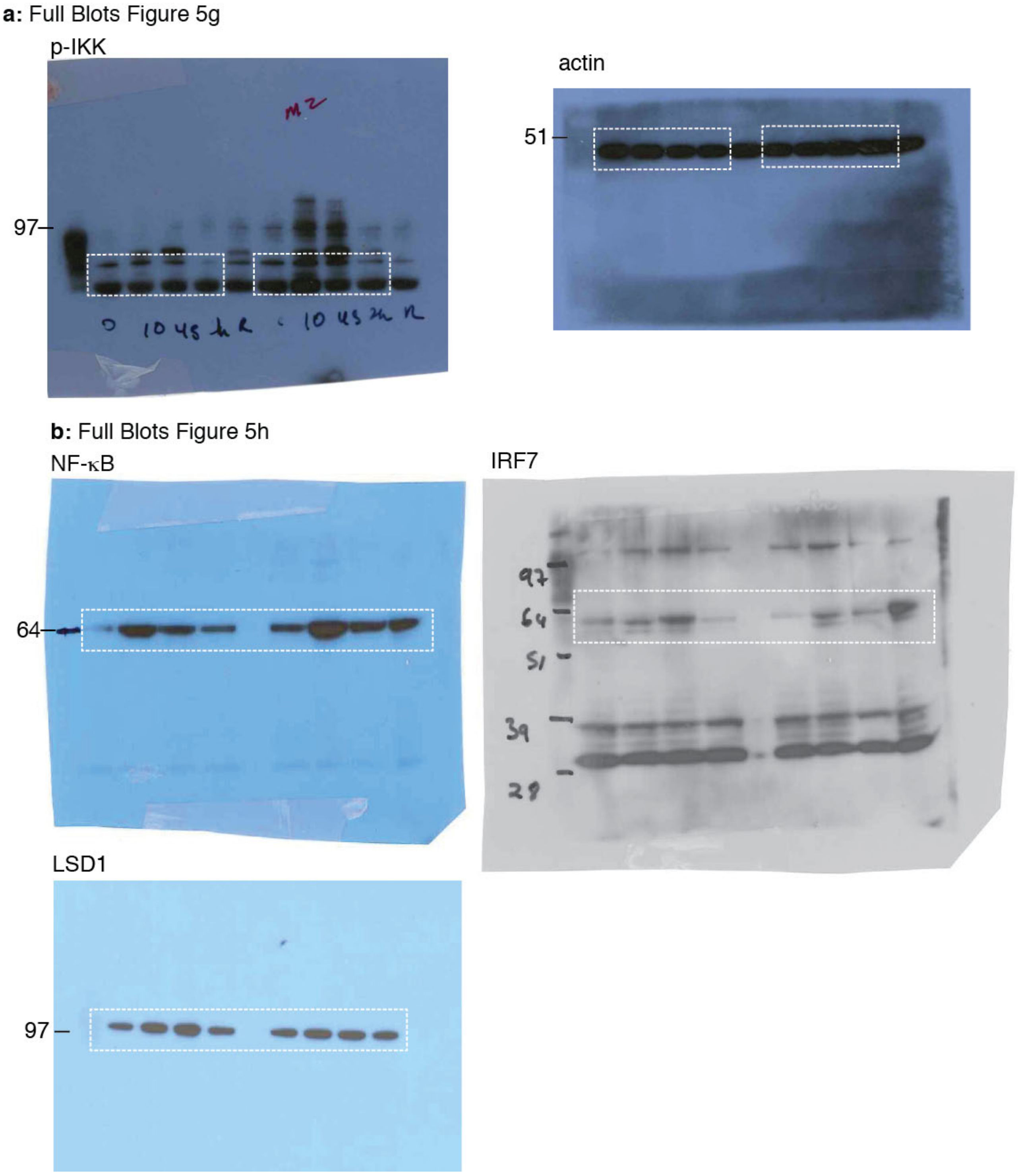

Supplementary Figure 7: Full Blots from Figure 5 (legend on next page)

c: Full Blots Figure 5k

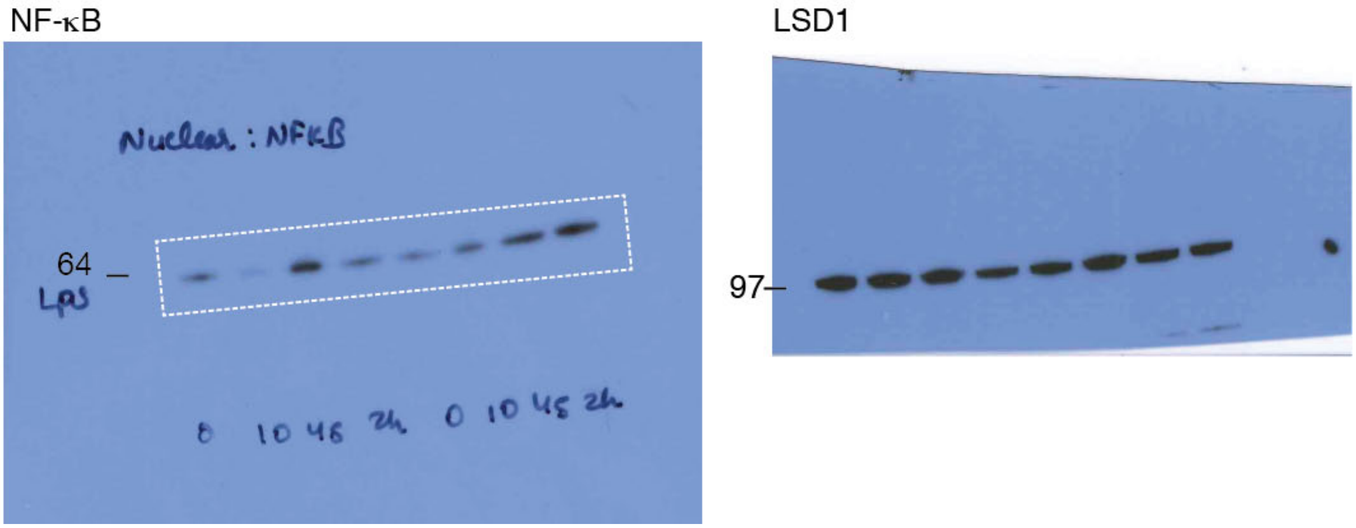

Supplementary Figure 7: Full Blots from Figure 5. Full western blots for p-Ikk (Fig. 5g) **(a)**, nuclear NF-κB, IRF7 and LSD1 (Fig. 5h) **(b)** and nuclear NF-κB and LSD1 (Fig. 5k) Dashed lines show regions presented in Figure 5. Molecular weight marker positions are indicated (kDa).

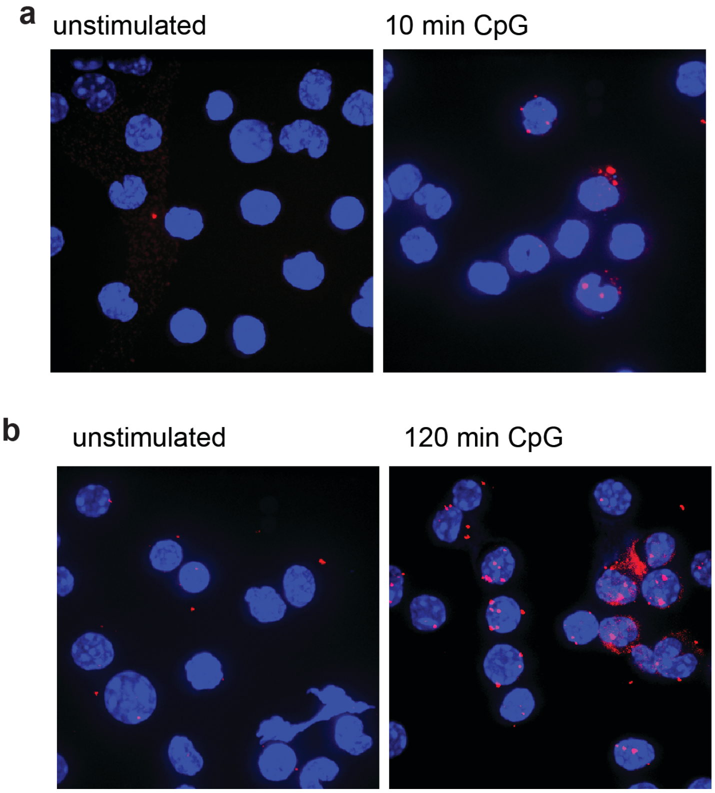

Supplementary Figure 8: Proximity Ligation Assay: (a-b) Proximity ligation assay (PLA) for αv integrin and TLR9 **(a)** and LC3 and TLR9 **(b)**. Images show sites of protein co-localization (red) and cell nuclei (blue). Images are representative from at least 10 images per condition used for quantification for histograms in Fig. 6e.

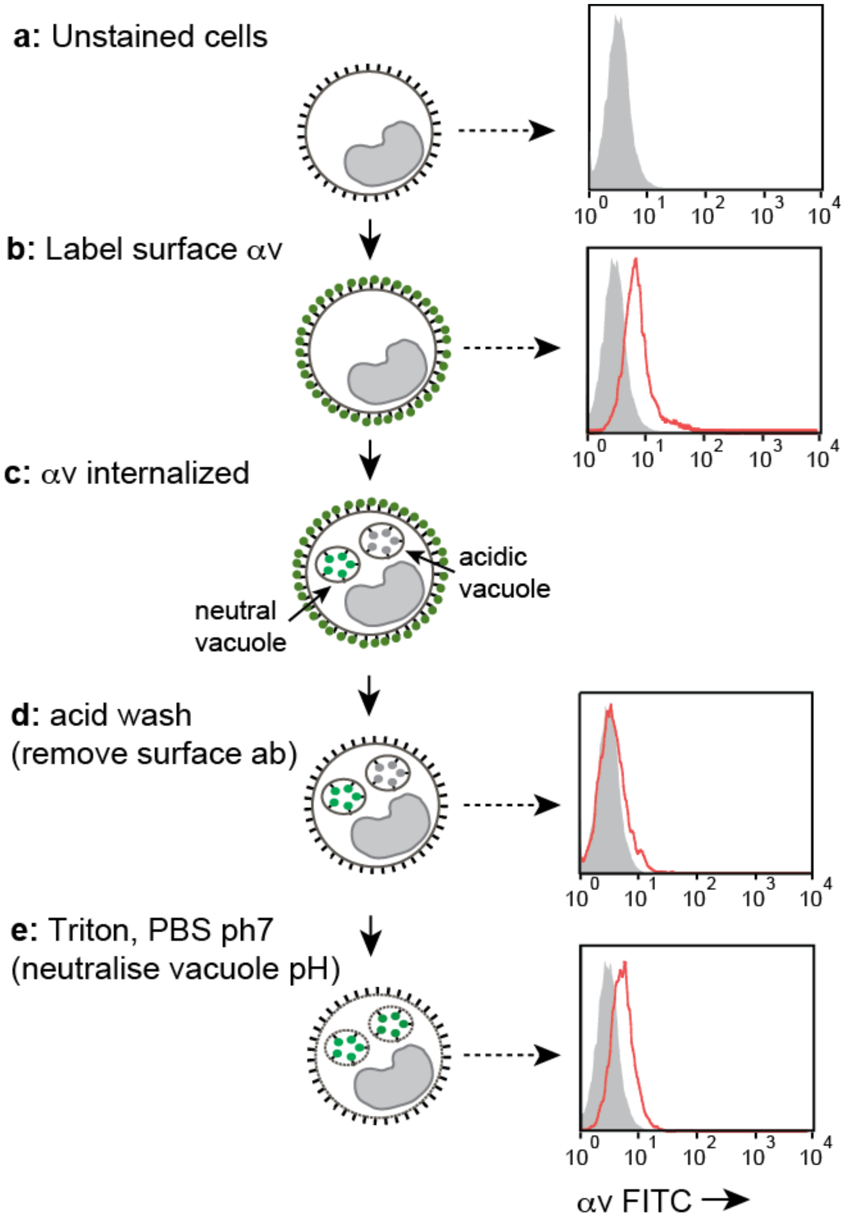

Supplementary Figure 9: Schematic of Integrin Internalization Assay. (a-b) Sorted MZ B cells were stained on ice with αv-FITC antibody to label surface αv integrins**. (c)** After staining cells were incubated at 37˚C in normal growth medium with or without CpG stimulation, during which time surface αv is internalized. Antibody internalized into neutral compartments will retain fluorescence, whereas internalization to acidic vacuoles will result in quenching of FITC fluorescence. **(d)** At 10-120 minutes after incubation, cells were washed with cold acidic wash buffer to remove and quench cell surface antibodies binding αv. Cells were analysed to measure αv internalized into neutral endosomal compartments. **(e)** Cells were re-suspended in neutral PBS with triton. This permeabilizes cell membrane and neutralizes pH in internal vacuoles, revealing FITC fluorescence in acidic compartments. Cells can be analyzed for total internalized integrin.

a: Full Blots Figure 7a

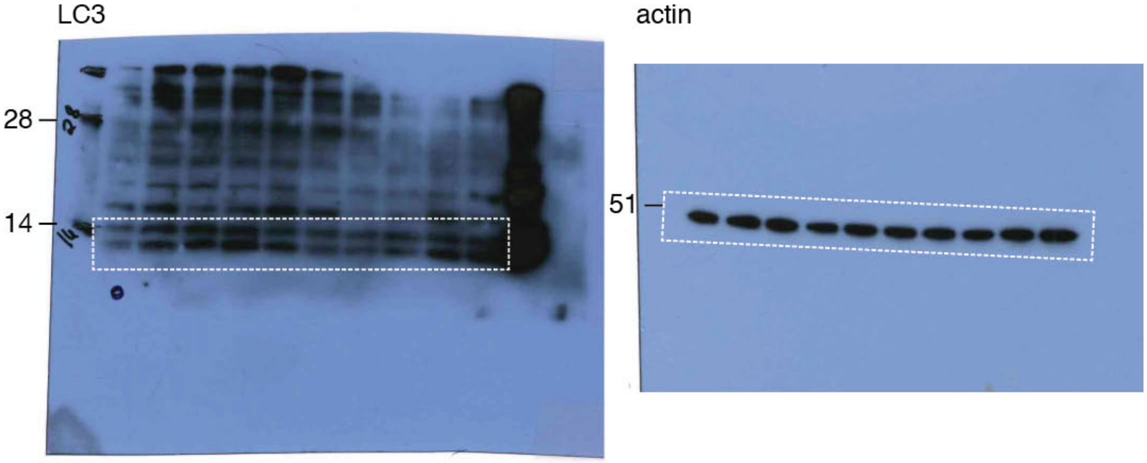

b: Full Blots Figure 7c

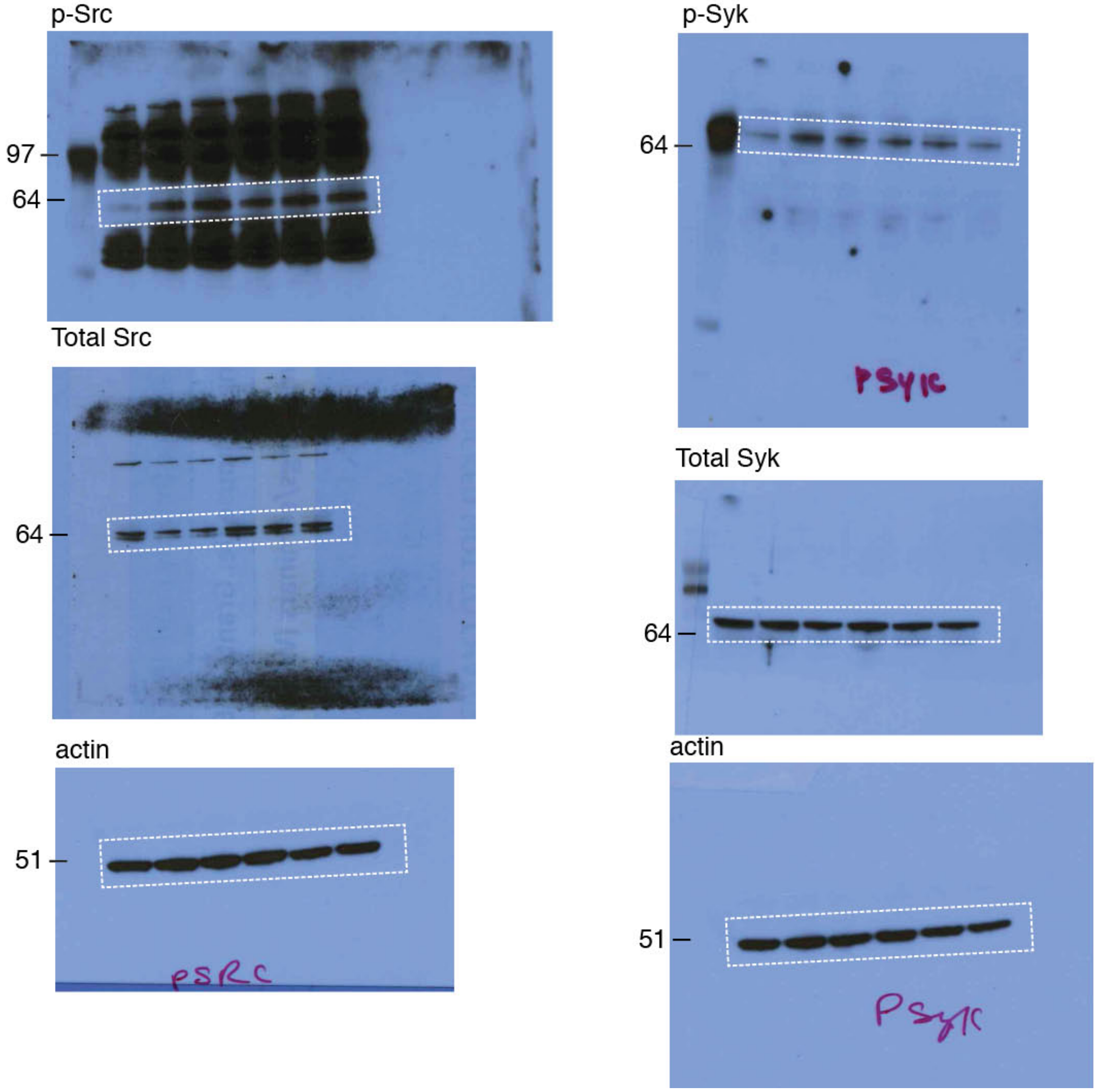

Supplementary Figure 10: (Continued over page)

c: Full Blots Figure 7d

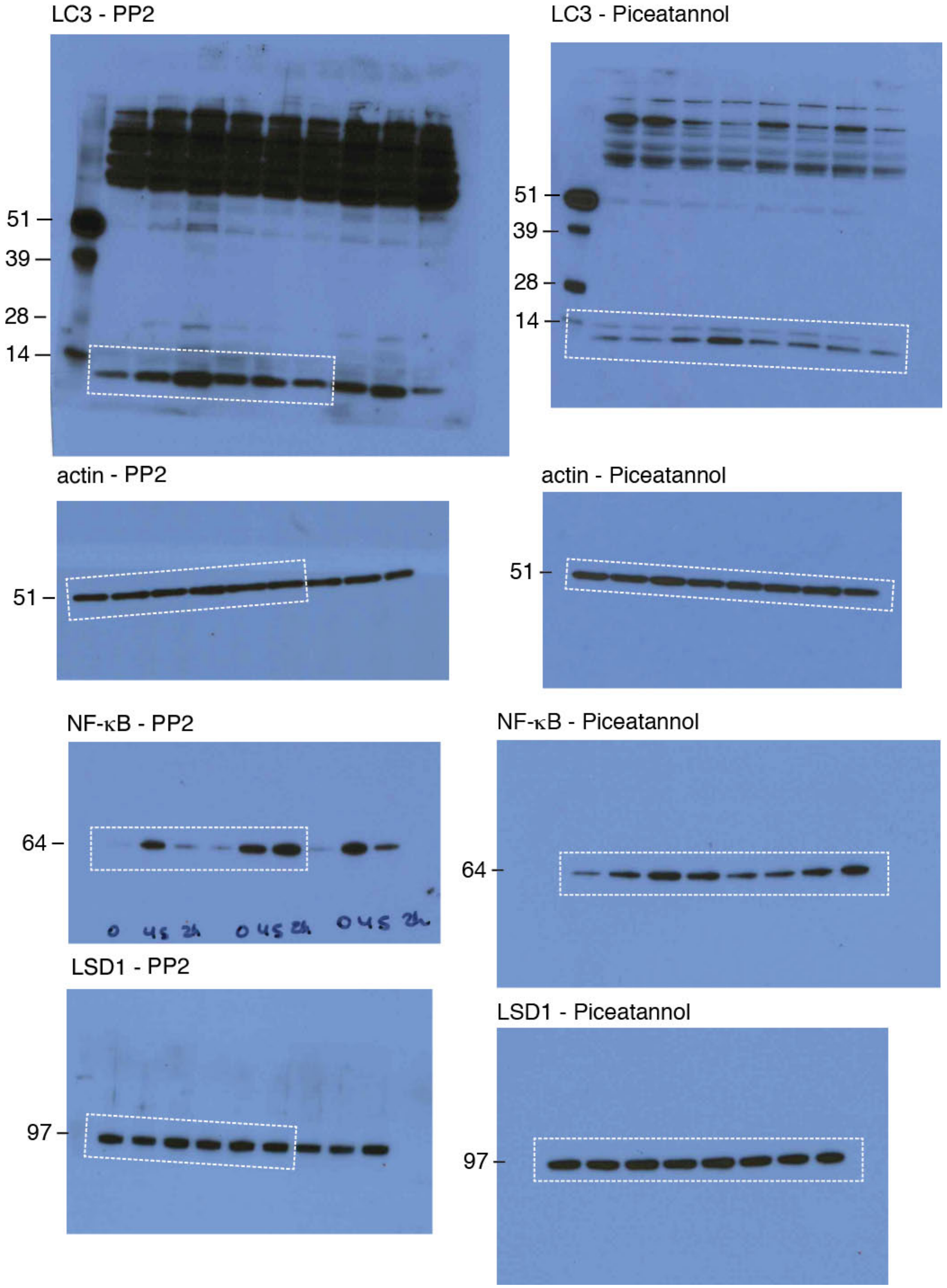

Supplementary Figure 10: (Continued over page).

d: Full Blots Figure 7f

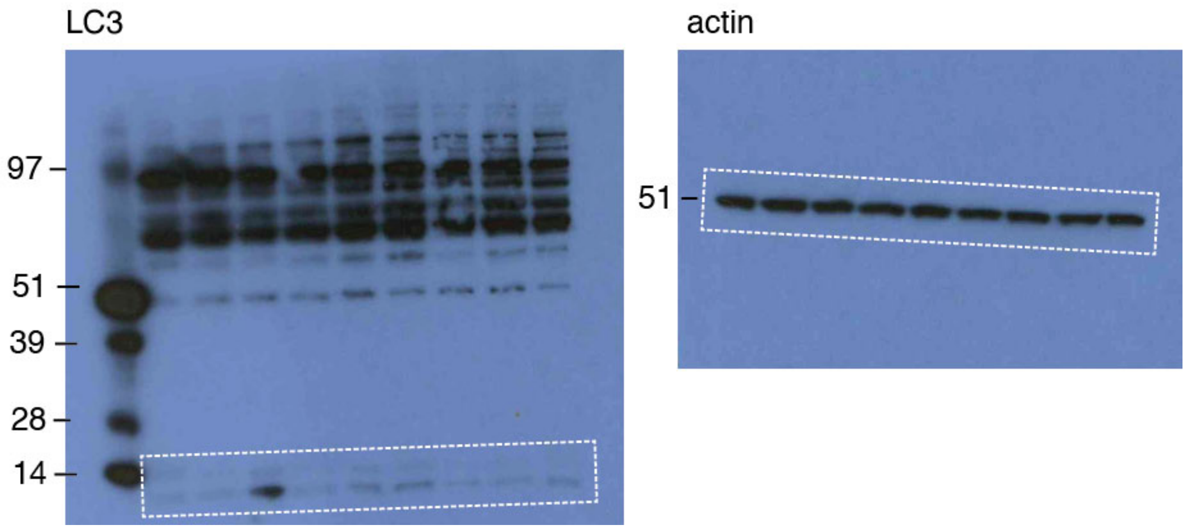

Supplementary Figure 10: Full blots for Figure 7. Full western blots for LC3 and actin (Fig. 7a) **(a)**; phospho- and total src and syk, with actin (Fig. 7c) **(b)**; LC3, actin, NF-κB and LSD1 (Fig. 7d) **(c)**; and LC3 and actin (Fig. 7f) **(d)**. Dashed lines show regions presented in Figure 7.

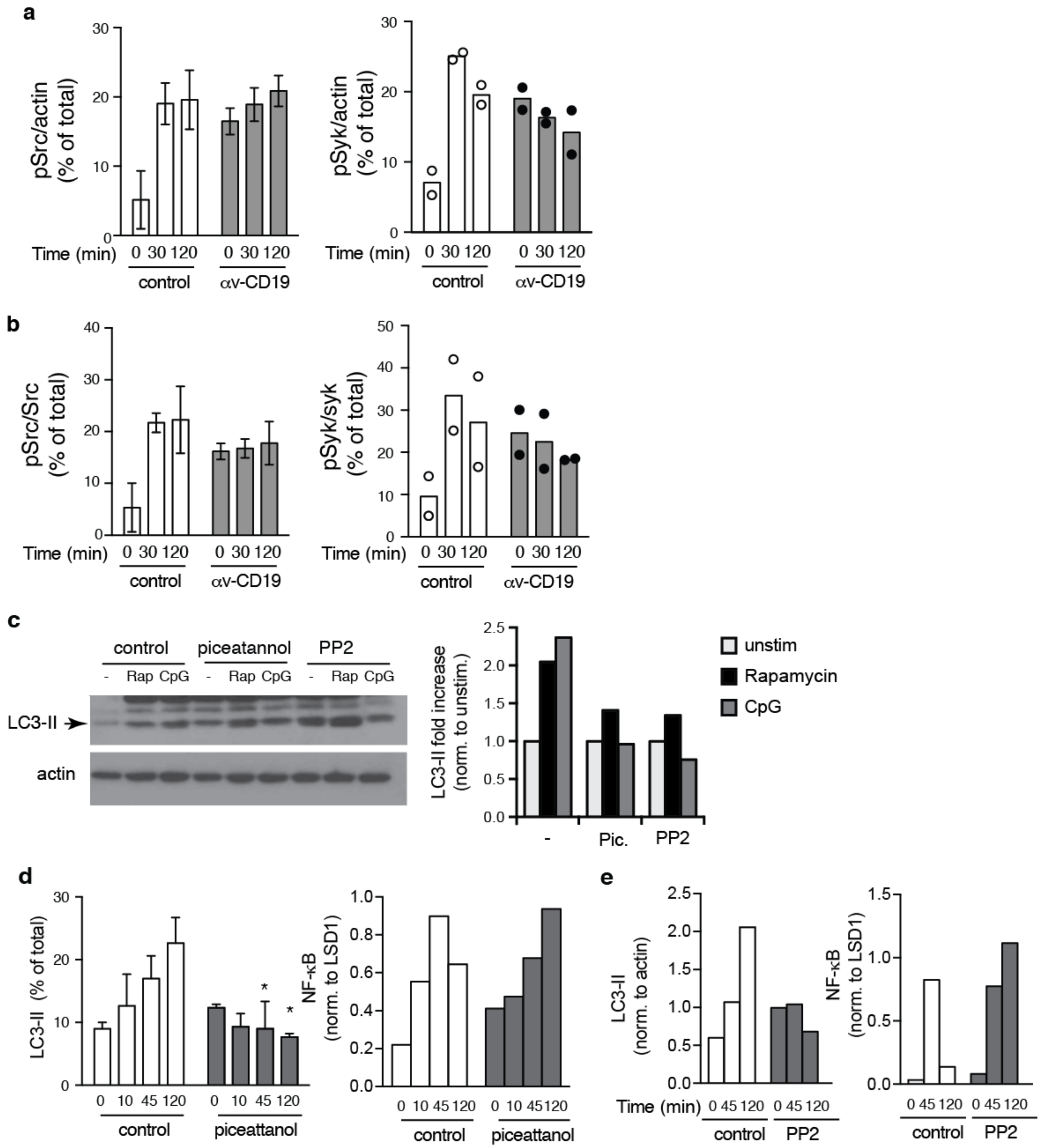

Supplementary Figure 11: Src/Syk regulate αv-dependent LC3 lipidation: (a-b) Quantification of western blot analysis of phospho-Src and phospho-Syk levels in sorted MZ B cells treated with CpG, including blots presented in Fig. 7c. Phospho-protein level were

normalized to either total kinase **(a)** or to the housekeeping protein actin **(b)** to correct for protein loading and levels. Corrected levels were then normalized by total signal in each experiment to allow analysis between experiments. Graphs show mean ± sd (n=3 independent experiments) or mean and individual experimental results. **(c)** Western blot analysis of LC3 and actin in sorted MZ B cells treated with kinase inhibitors and CpG or rapamycin. Arrow indicates position of LC3-II band. Graph shows quantified levels of LC3-II corrected for loading using actin staining, and then normalized to unstimulated cells to allow analysis of LC3-II induction. **(d)** Quantification of western blot analysis from Fig. 7d. Graphs show mean ± sd for experiments where multiple blots can be combined, or quantification of a representative blot. *, $p<0.05$, Student's t-test.

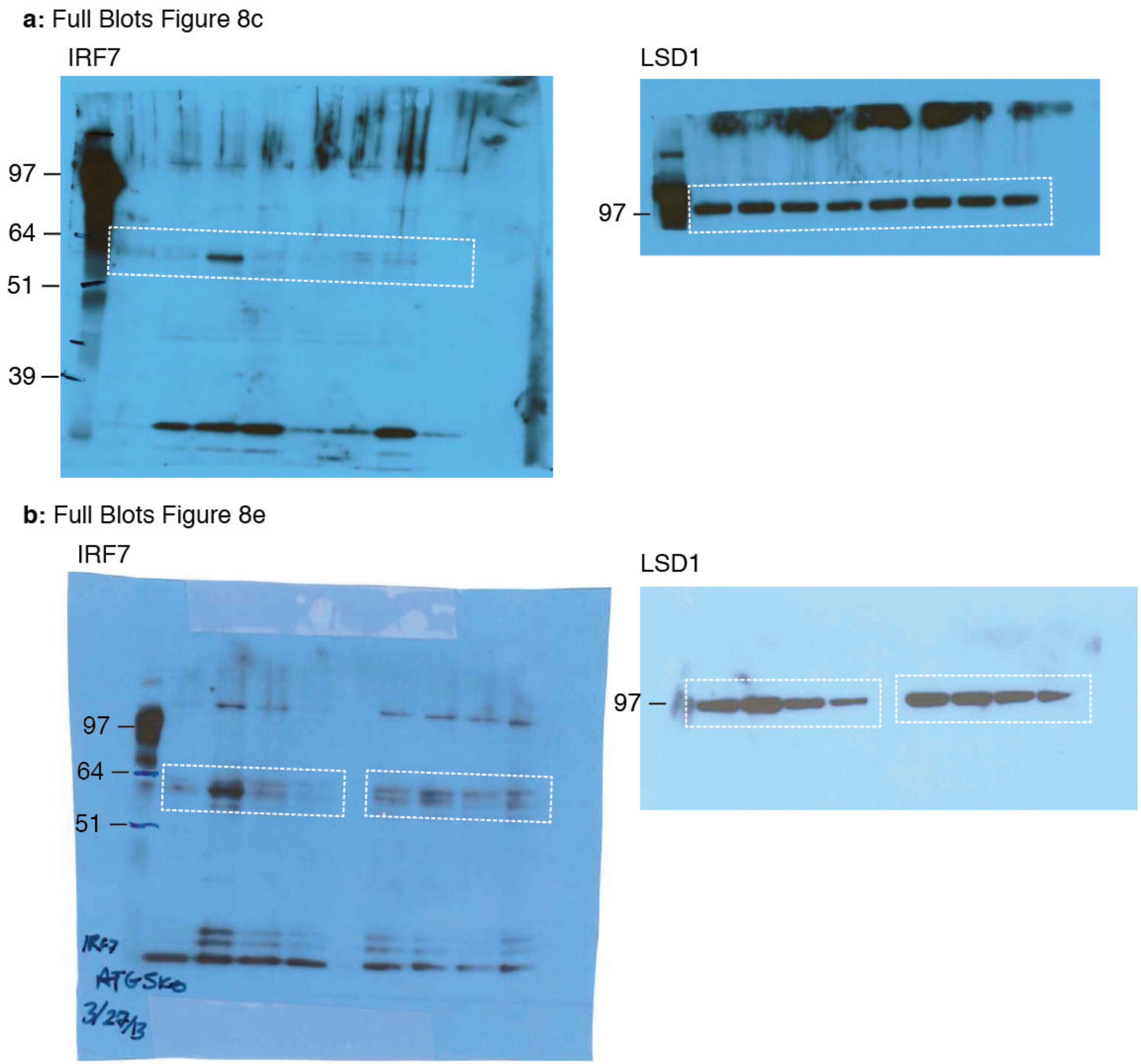

Supplementary Figure 12: Full blots for Figure 8. Full western blots for IRF7 and actin for Figs. 8c **(a)** and 8e **(b)**. Dashed lines show regions presented in Figure 8.

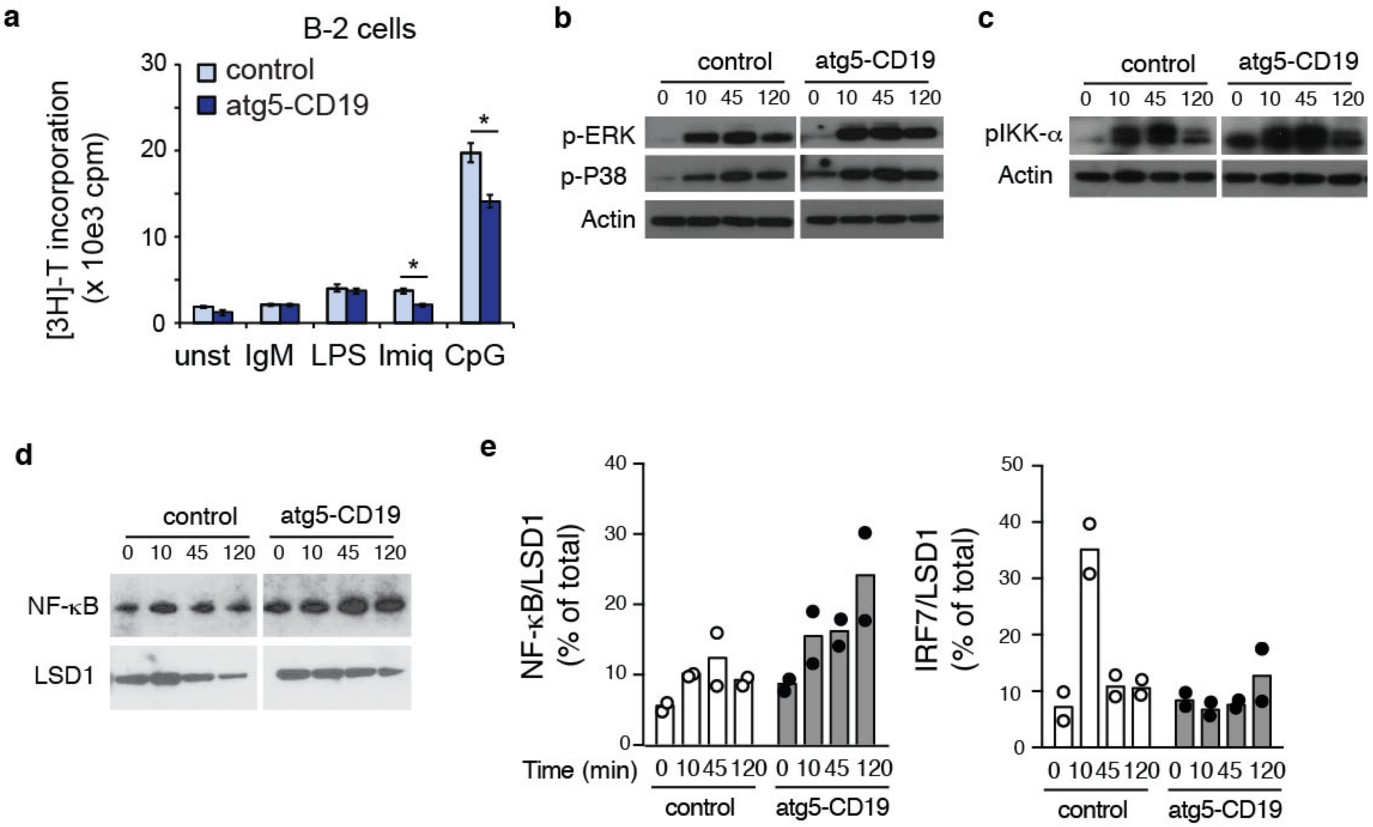

Supplementary Figure 13: Increased B cell responses to TLR stimulation in atg5-CD19 mice. (a) Proliferation of sorted peritoneal B-2 cells from atg5-CD19 conditional knockout mice in response to TLR ligands. Each point is mean ± s.d of n=3 replicates. Similar results were seen in 3 independent experiments. *, significantly different $p < 0.05$, Student's t-test. **(b-d)** Western blot analysis of signaling components downstream of TLR9 in sorted MZ B cells isolated from atg5-CD19 mice, stimulated with CpG DNA for indicated times (min). Blots show cytoplasmic phosphorylated MAP-kinases **(b),** phosphorylated IKKα **(c)** and nuclear NF-κB **(d)** as well as actin or LSD1 staining to confirm equivalent protein loading. **(e)** Quantification of NF-κB and IRF7 (from **(d)**, Fig. 8e and additional blots). NF-κB and IRF7 corrected for gel loading using LSD1 were then normalized to total signal for each experiment to allow comparison between experiments. Bars indicate mean and individual values for 2 independent experiments.

a: Full Blots from Supp Fig 13 b

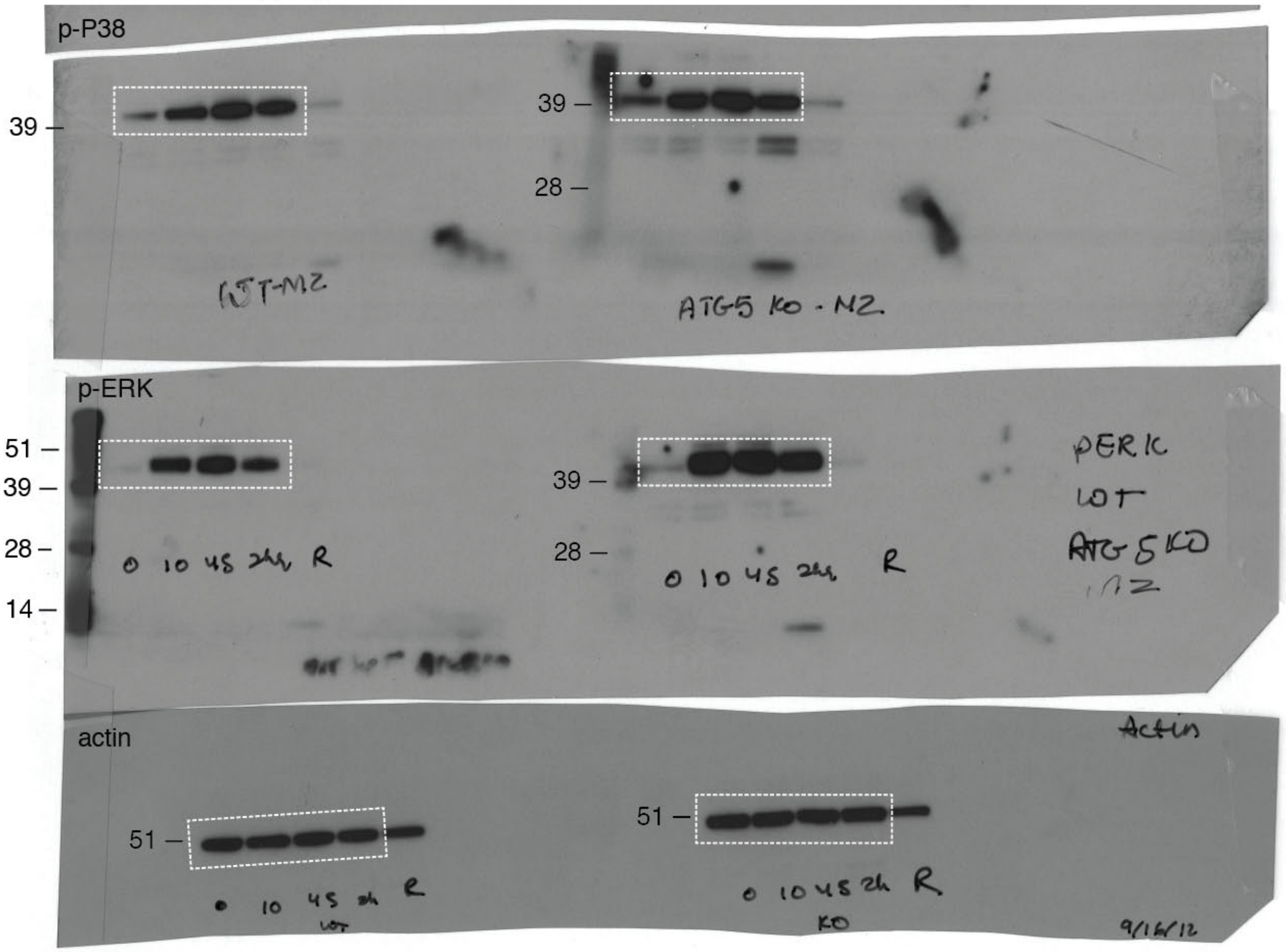

b: Full Blots from Supp Fig 13 c

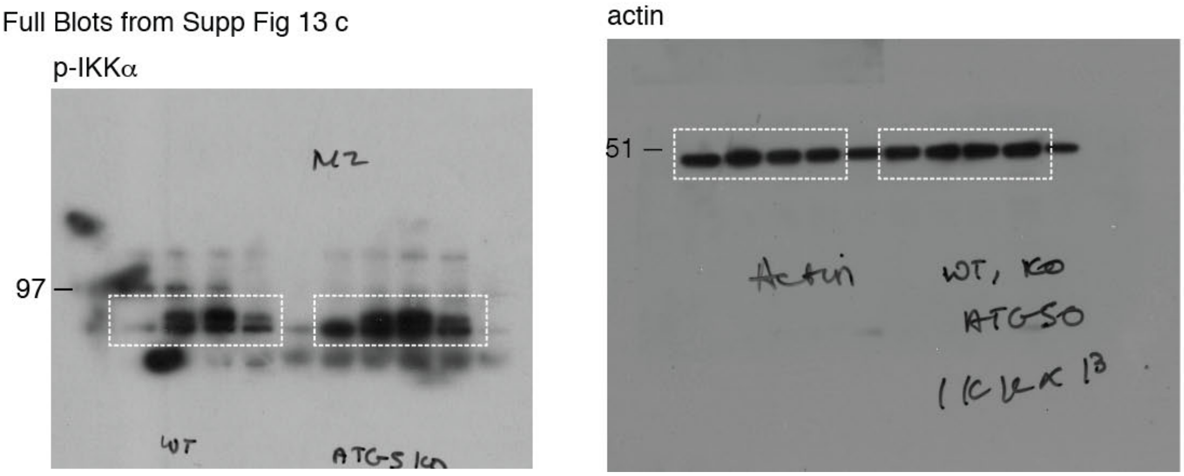

Supplementary Figure 14: Full blots for Supplementary Figure 13: Full western blots for p-P38, p-ERK and actin for Supp. Fig. 13b **(a),** and p-IKK and actin for Supp. Fig. 13c **(b)**. Dashed lines show regions presented in Supp. Fig. 13.

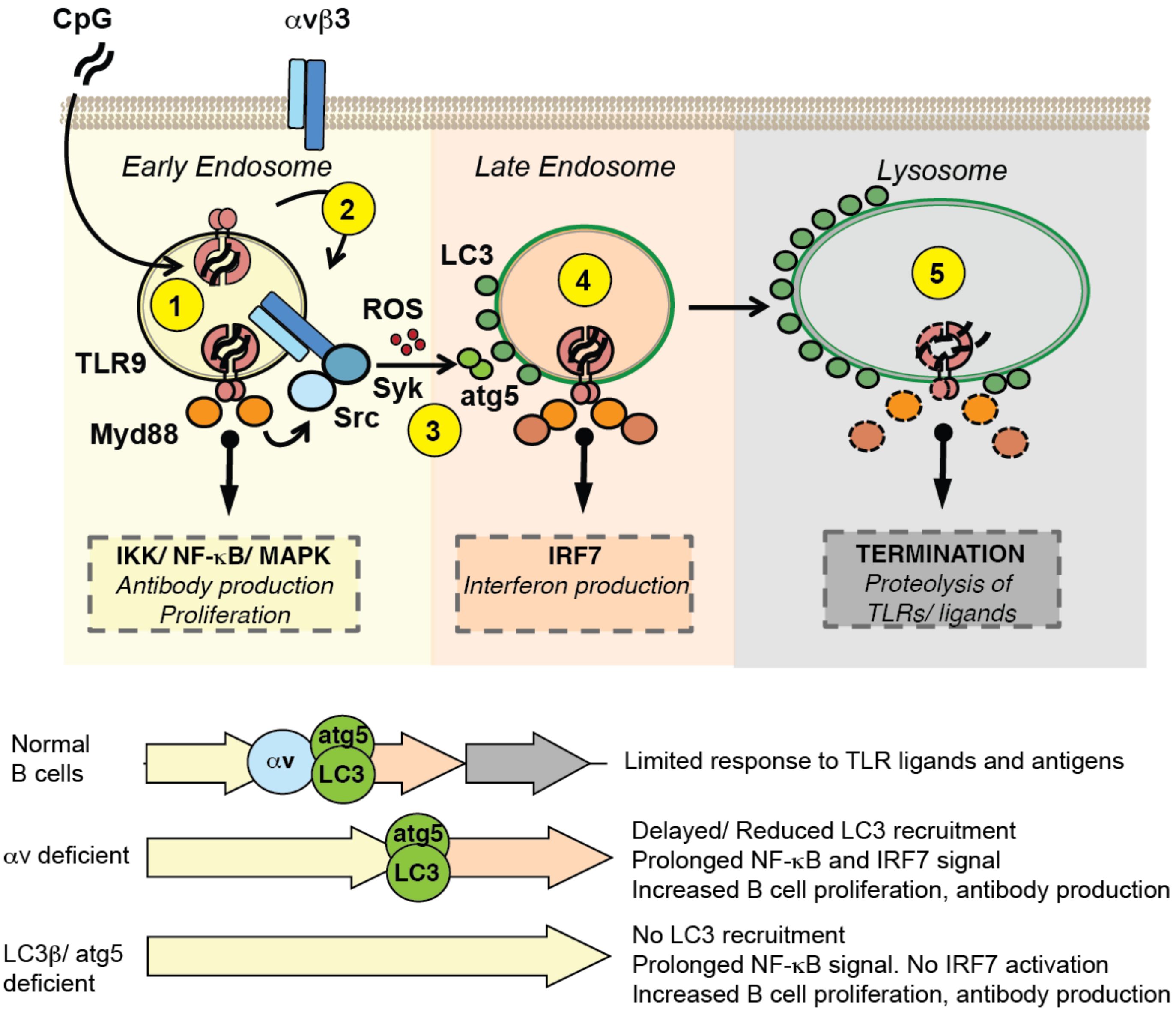

Supplementary Figure 15: Schematic model for αv-mediated regulation of TLR signaling in B cells: (1) CpG DNA encounters TLR9 in endosomes and initiates TLR signaling. **(2)** TLR signaling triggers internalization of surface αv to the TLR-containing early endosome. **(3)** αv mediates phosphorylation of Src and Syk, which cause lipidation of LC3 and recruitment to the endosomal membrane though a mechanism requiring ROS. **(4)** LC3 recruitment causes transition of TLR9 to a late endosome, which is permissive for activation of IRF7 transcription factors rather than NF-κB. **(5)** Late endosomes then fuse with lysosomes, leading to degradation of TLR ligands and signaling complexes, resulting in termination of TLR signaling. Through this mechanism, αv promotes the transit of TLRs from NF-κB-competent endosomes, through IRF7 signaling compartments, to sites where TLR signaling is terminated. In the absence of αv, this process is delayed, leading to prolonged NF-κB and later IRF7 signaling. In the absence of LC3b or atg5, TLRs cannot transition to the late endosome, causing prolonged NF-κB activation.
